# Effect of pH and concentration on the chemical stability and reaction kinetics of thiamine mononitrate and thiamine chloride hydrochloride in solution

**DOI:** 10.1186/s13065-021-00773-y

**Published:** 2021-08-12

**Authors:** Adrienne L. Voelker, Lynne S. Taylor, Lisa J. Mauer

**Affiliations:** 1 Department of Food Science, Purdue University, 745 Agriculture Mall Drive, West Lafayette, Indiana 47907 USA; 2 Department of Industrial and Physical Pharmacy, Purdue University, 575 Stadium Mall Drive, West Lafayette, Indiana 47907 USA

**Keywords:** Thiamine, Vitamin B_1_, Chemical stability, Degradation, pH, Reaction kinetics, Activation energy

## Abstract

Thiamine (vitamin B_1_) is an essential micronutrient in the human diet, found both naturally and as a fortification ingredient in many foods and supplements. However, it is susceptible to degradation due to heat, light, alkaline pH, and sulfites, among effects from other food matrix components, and its degradation has both nutritional and sensory implications as in foods. Thiamine storage stability in solution was monitored over time to determine the effect of solution pH and thiamine concentration on reaction kinetics of degradation without the use of buffers, which are known to affect thiamine stability independent of pH. The study directly compared thiamine stability in solutions prepared with different pHs (3 or 6), concentrations (1 or 20 mg/mL), and counterion in solution (NO_3_^−^, Cl^−^, or both), including both commercially available salt forms of thiamine (thiamine mononitrate and thiamine chloride hydrochloride). Solutions were stored at 25, 40, 60, and 80 °C for up to one year, and degradation was quantified by high-performance liquid chromatography (HPLC) over time, which was then used to calculate degradation kinetics. Thiamine was significantly more stable in pH 3 than in pH 6 solutions. In pH 6 solutions, stability was dependent on initial thiamine concentration, with the 20 mg/mL thiamine salt solutions having an increased reaction rate constant (k_obs_) compared to the 1 mg/mL solutions. In pH 3 solutions, k_obs_ was not dependent on initial concentration, attributed to differences in degradation pathway dependent on pH. Activation energies of degradation (E_a_) were higher in pH 3 solutions (21–27 kcal/mol) than in pH 6 solutions (18–21 kcal/mol), indicating a difference in stability and degradation pathway due to pH. The fundamental reaction kinetics of thiamine reported in this study provide a basis for understanding thiamine stability and therefore improving thiamine delivery in many foods containing both natural and fortified thiamine.

**Supplementary Information:**

The online version contains supplementary material available at 10.1186/s13065-021-00773-y.

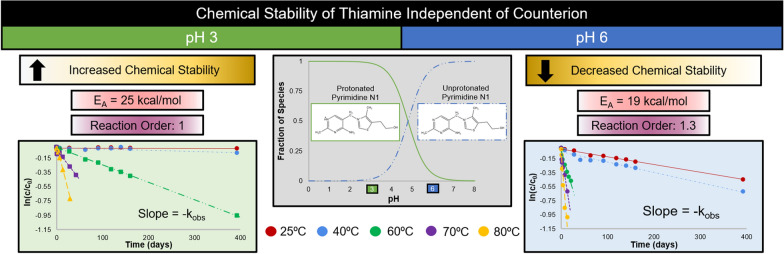

## Introduction

**Fig. 1:**
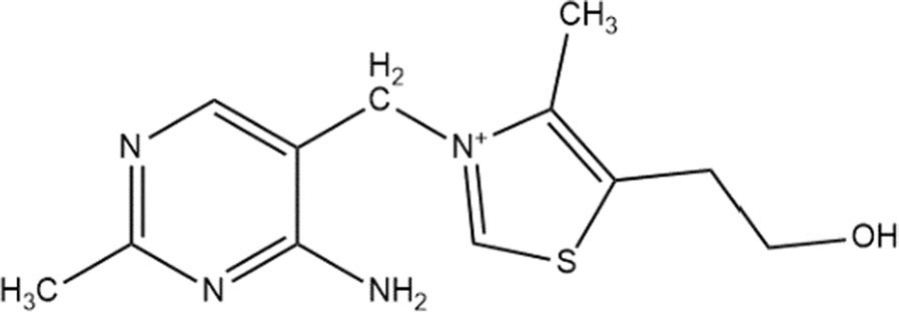
Chemical structure of thiamine

Thiamine (vitamin B_1_; Fig. 1) was the first vitamin to be characterized [1]. It is an essential micronutrient in the human diet, with a Recommended Dietary Allowance (RDA) and Daily Value (DV) of 1.2 mg/day in the United States [2, 3]. It is found naturally in foods, such as grains, legumes, nuts, and meats [4]. Thiamine acts as a precursor for a coenzyme in the metabolism of carbohydrates, branched-chain amino acids, and lipids, and plays major roles in muscle contraction and in the nervous system [2, 5]. While grains are the main source of thiamine in the diet, the thiamine is mostly located in the germ and the bran, the outer layers of the kernel, so thiamine content is reduced by 89% during the refining process [6, 7]. For this reason, thiamine deficiency is a concern in both developed and developing countries. A lack of a nutritious diet is the main cause of thiamine deficiency in developing countries, especially when the main dietary component is an unfortified grain, e.g., polished rice [8]. In developed countries where malnutrition or lack of fortification is less of a concern, deficiency is still common in certain groups of people, including alcoholics, people with HIV/AIDS, and people on diets that avoid fortified grains, such as those with Celiac’s disease [5, 9].

Two salt forms of thiamine [thiamine mononitrate (TMN) and thiamine chloride hydrochloride (TClHCl)] are commonly added to foods as enrichment or fortification supplements. Although this has substantially reduced thiamine deficiency in developed countries, up to 84% of thiamine in foods can still be lost during cooking or processing due to the instability of the vitamin [10]. Thiamine is sensitive to heat, alkali, salts, oxygen, and sulfites [11–14]. Previous studies have shown that TMN and TClHCl have different activation energies (E_a_) of degradation both in the solid state (26.3 and 22.4 kcal/mol, respectively) and in solution (21 and 32 kcal/mol, respectively in 10 mg/mL solutions) [15, 16]. Differences in E_a_ suggest that the degradation pathway differs between the two salt forms, which also has sensory implications due to sulfur-containing degradation products [16–18]. However, the salt form of thiamine is dissociated when dissolved in solution, so it was proposed that the difference in E_a_ in solution and therefore difference in degradation pathway was due to the pH of the solution rather than the stability of the salt form itself [16, 19, 20].

Thiamine degradation has been reported to be a pseudo-first order reaction and therefore dependent on concentration [11, 21, 22]. It is known to be more stable in acidic conditions, specifically below a pH of 6.0 due to thiamine’s food-relevant pK_a_ of 4.8, wherein the less stable thiamine species (unprotonated pyrimidine N1) is the predominant species above pH 6.0 [14, 19, 23]. However, pH-dependent thiamine stability is most often studied by employing the use of common buffer systems despite studies that have shown both type and concentration of buffer salts to affect thiamine degradation independent of pH [14, 19, 24, 25]. Although many kinetic studies on thiamine degradation have been published [26–28], more research is needed to understand the true effect of pH on the long-term stability of thiamine in solution at food-relevant temperatures without the unintended effect of common buffer salts or protective action of other food components.

It was hypothesized that the salt form of vitamin (TMN vs. TClHCl) and, in effect, counterion in solution (NO_3_^−^ vs. Cl^−^) would not affect the stability of thiamine, but rather pH of solution and thiamine concentration would play the most significant roles in dictating thiamine stability. Thus, the objectives of this study were to: (1) investigate the impacts of thiamine concentration and solution pH on thiamine stability in the absence of common buffer salts, (2) compare the effect of counterion of thiamine salts in solution on thiamine stability, and (3) calculate and compare reaction kinetics of pH-dependent thiamine degradation. The results of this study can be used to improve the nutritional quality of food products by better understanding the role of pH on thiamine stability.

## Materials and methods

### Materials

Two salt forms of thiamine were used in this study: thiamine mononitrate, C_12_H_17_N_4_OS·NO_3_ (TMN) (Spectrum Chemical Mfg. Corp., New Brunswick, NJ) and thiamine chloride hydrochloride, C_12_H_17_ClN_4_OS·HCl (TClHCl) (Fisher Scientific, Fair Lawn, NJ). Nitric acid (HNO_3_) (J.T. Baker, Center Valley, PA), hydrochloric acid (HCl) (Acros Organics, Fair Lawn, NJ), and sodium hydroxide (NaOH) (Sigma-Aldrich, St. Louis, MO) were used to adjust the pH of thiamine solutions. For use in high performance liquid chromatography (HPLC), HPLC grade acetonitrile and trifluoroacetic acid (TFA) were obtained from Fisher Scientific. All water used throughout the study was deionized and purified using a Barnstead E-pure ultrapure water purification system with a resistivity greater than 17.5 MΩ·cm at 25 °C (ThermoScientific, Waltham, MA).

### Sample preparation

Previous studies have investigated the effect of thiamine concentration on stability in solution [16]. It was found that while TMN stability was influenced by concentration, TClHCl was less affected, which was attributed to pH, wherein the solution pHs (approximately 6 and 3, respectively) were unadjusted and dependent on thiamine salt concentration. Therefore, to understand the impact of solution pH and concentration on thiamine stability a series of TMN and TClHCl solutions were prepared at two pHs and two concentrations: pHs 3 and 6 at thiamine concentrations 1 and 20 mg/mL. The samples were prepared on a weight basis rather than by molar concentration. Although TMN and TClHCl have slightly different molecular weights, such that the actual concentrations of dissociated thiamine were 0.81 and 16.2 mg/mL in 1 and 20 mg/mL TMN solutions and 0.79 and 15.7 mg/mL in 1 and 20 mg/mL TClHCl solutions, degradation calculations were done using percent remaining, which accounts for the differences in molecular weights. Although the concentrations used in this study were higher than those found in foods, the higher concentration was used to enable more accurate thiamine analysis.

TMN solutions were adjusted to pH 3 and 6 using HNO_3_ and NaOH. Nitric acid was used to adjust TMN solutions to limit counterions to only nitrate. TClHCl solutions were adjusted to the same pHs using HCl and NaOH. Hydrochloric acid was used to adjust TClHCl solutions to limit counterions to only chloride. Solutions were also prepared with the alternate acid (TMN with HCl and TClHCl with HNO_3_) to determine if counterion (NO_3_^−^ vs. Cl^−^) influenced thiamine degradation patterns. A previous study by our group was completed in which solution pHs were not adjusted [16]. This data was used as a control point for comparison. All solutions (10 mL) were prepared in triplicate in 20 mL amber glass scintillation vials with PE cone-lined phenolic caps and sealed with duct tape to prevent evaporation.

### Sample storage

**S**olutions were stored at 5 temperatures: 25, 40, 60, 70, and 80 °C using a method by Voelker et al. [16] to investigate the effect of temperature on chemical stability. These temperatures were chosen based on conditions that may be experienced in the food industry, specifically during storage, processing, or accelerated shelf-life testing, and for temperature-dependent reaction kinetics calculations. The 25 °C condition was maintained using a temperature-controlled room. Samples were kept in 40 °C, 60 °C, and 70 °C environments using Forma Scientific water-jacketed incubators (Thermo Fisher Scientific Inc., Marietta, OH). The 80 °C temperature was maintained using a digital heatblock (VWR International, Radnor, PA). Temperature was confirmed over the duration of the study using thermometers. Depending on temperature and pH, solutions were stored in controlled temperature environments for up to 1 year. Samples were analyzed in triplicate for percent thiamine remaining at a minimum of 5 selected timepoints.

### Vitamin quantification

The chemical stability of thiamine in solution was measured in accordance with an adaptation of AOAC method 942.23 for quantification of thiamine [29]. Reverse-phase HPLC (Waters Corp. Milford, MA) using a gradient method with 0.1% TFA in water (v/v) and acetonitrile as the mobile phases, A and B, respectively, was used in accordance with our previous study [16]. Briefly, a Waters 2690 Separations Module and a Waters 2996 Photodiode Array (PDA) detector were used with a Waters XTerra RP-C_18_ column and a wavelength scan of 235–400 nm. The gradient method was as follows: 100/0 at 0 min, 97/3 at 4 min (linear), 90/10 at 6 min (linear), 100/0 at 10 min (linear), and 100/0 at 15 min. Prior to analysis, solutions were cooled in an ice bath, and diluted with the 0.1% TFA in water mobile phase to an estimated thiamine concentration of 500 ppm, or 0.5 mg/mL (assuming no degradation). Standard curves of TMN and TClHCl (R^2^ > 0.999) were prepared using the area under the analyte peak to calculate thiamine concentration of samples on each day of analysis using a concentration range of 10 ppm to 1000 ppm. Integration of the analyte peak was performed at 254 nm.

### Reaction kinetics

Reaction kinetics were calculated to monitor the kinetics of thiamine degradation as affected by pH and counterion in solution using similar calculations to our preceding study [16]. Previous work has shown thiamine degradation to be a pseudo first-order reaction [11, 16, 21], and under this assumption, the kinetic rate constants (*k*) were calculated using the following first-order equation:

1 $$ln\frac{x}{{x_{0} }} = - kt$$

where *x* is the concentration of thiamine at time *t* (days), *x*_*0*_ is the initial thiamine concentration, and *k* is the reaction rate constant (days ^−1^).

The Arrhenius equation was used to describe temperature dependence of *k*:

2 $$k = Ae^{{\frac{{ - E_{a} }}{RT}}}$$

where *k* is the reaction rate constant (days^−1^), *A* is the frequency factor of collision (which can be eliminated when *k* is known at two or more temperatures, as is the case in this study), *E*_*a*_ is the activation energy (kJ/mol), *R* is the gas constant (8.3145 J/mol·K), and *T* is temperature (K). Our previous study [16] found that linear degradation patterns were generally lost when 40% or less of thiamine remained due to side-reactions of the degradation products, so calculations only included data up to that point. The t_90_ values were also calculated to indicate the time at which 90% of the initial thiamine concentration remained (10% had degraded).

### pH measurement

The pH of all samples was measured over time to monitor how pH changed from the original pH 3 or 6 value over the duration of the experiment. An Orion pH probe (ThermoScientific) that had been calibrated using pH 1.68, 4.01, and 7.00 calibration standards obtained from ThermoScientific was used in this study. Solution pHs were measured at least 3 times over the duration of the experiment, including a measurement at the first HPLC timepoint (following day 0), at least one midpoint, and the final timepoint of HPLC analysis. Solution pHs were measured in duplicate.

### Statistical analysis

All samples were prepared and analyzed by HPLC in triplicate for each timepoint of analysis, and single variable ANOVA using SAS 9.4 (SAS Institute, Cary, NC) with Tukey’s post-hoc test for multiple comparisons (α  =  0.05) was used to determine significant differences in: (1) percent thiamine remaining between the initial solution and the partially degraded sample over time, and (2) percent thiamine remaining between sample types at the same time point. Regression analysis was used to determine standard error of the slopes used to calculate k_obs_ and E_a_ values, and t_90_ values were calculated to indicate time when 90% of the initial thiamine remained. Single-variable ANOVA was also used to determine significant differences in pH.

## Results and discussion

### Chemical stability of thiamine in pH 6 solutions

**Fig. 2:**
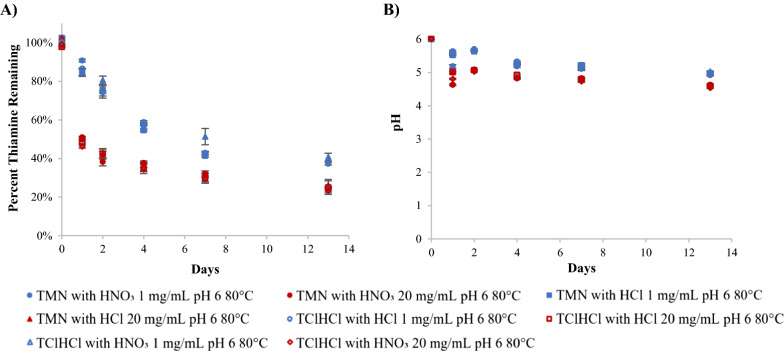
Chemical behavior of thiamine in pH 6 solutions with varying counterion (NO_3_^−^ or Cl^−^) and concentration (1 or 20 mg/mL) at 80 °C over time including: **A** degradation profiles and **B** pH profiles

**Fig. 3:**
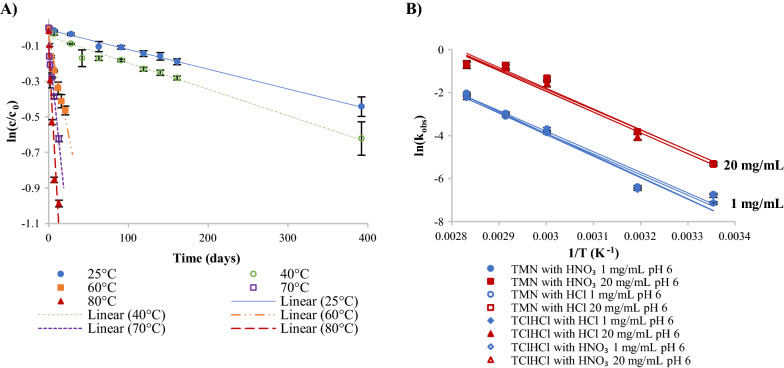
Reaction kinetics of thiamine degradation in pH 6 thiamine solutions: **A** first-order degradation regression lines of 1 mg/mL TMN solutions adjusted to pH 6 with HNO_3_ and NaOH at temperatures from 25 to 80 °C; and **B** Arrhenius plots used to calculate temperature-dependent activation energy for thiamine degradation in 1 and 20 mg/mL thiamine solutions adjusted to pH 6 with HNO_3_ or HCl and NaOH from 25 to 80 °C

Thiamine is often reported to become less stable at a pH of 6 compared to more acidic conditions [14, 21, 30], so this study analyzed thiamine stability at that pH. Both temperature and concentration were found to significantly (p  <  0.05) affect the stability of thiamine in pH 6 solutions, with higher temperatures and higher concentrations causing more degradation (Figs. 2A, 3A). Generally, the percent of thiamine remaining in 1 mg/mL solutions at the same temperature and day of analysis were not statistically different from one another (p > 0.05). Similarly, the percent of thiamine remaining in 20 mg/mL solutions were not statistically different from one another; however, 1 mg/mL solutions had significantly more thiamine remaining than 20 mg/mL solutions at the same timepoint (p  <  0.05) (Additional file 1: Tables S1, S2). This can be exemplified by the percent thiamine remaining on day 2 following storage at 80 °C. The TMN with HNO_3_, TMN with HCl, TClHCl with HNO_3_, and TClHCl with HCl solutions (all 1 mg/mL) contained 74, 75, 81, and 74% thiamine, respectively, while the same sample types at the higher 20 mg/mL concentration contained 38, 42, 42, and 42% thiamine, respectively. Thus, all 1 mg/mL samples contained significantly (p  <  0.05) more thiamine on day 2 than any of the 20 mg/mL samples. This in agreement with what was found in a previous study at similar pHs, but with the unmodified pH only dependent on concentration and thiamine salt form [16].

An example of a typical degradation profile is shown in Fig. 2A, in which thiamine degraded in an exponential manner. All percent thiamine remaining data for all temperatures, concentrations, and counterions are provided in the additional files (Additional file 1: Tables S1, S2). The pH was also monitored over the duration of the experiment, and an example of a typical pH change over time is shown in Fig. 2B. The pH of all samples that were initially at pH 6 remained above 4.5 following storage at all temperatures for the duration of the study, with many samples remaining above a pH of 5. The largest drop in pH came at the first timepoint of analysis, with smaller decreases coming at each subsequent timepoint. The 20 mg/mL solutions dropped to lower pHs than 1 mg/mL solutions. The change in pH was presumably caused by the degradation products present in solution following partial degradation. This same lowering of pH over time was also seen in our previous study [16]. Overall, the lowering of pH over the duration of the study was not considered to affect the thiamine stability due to the high correlations of linear regressions used to calculate reaction kinetics even as pH decreased. Tables including all pH data over time for all temperatures, concentrations, and counterions are also provided in the additional files (Additional file 1: Tables S3, S4).

### Degradation kinetics of thiamine in pH 6 solutions

Due to the apparent first-order reaction behavior observed in the pH 6 solutions, Eq. 1 was used to calculate the observed reaction rate constant (k_obs_) for each sample preparation. High correlations were observed for all linear regressions of the natural log of percent thiamine remaining vs. time (R^2^  =  0.82–0.99). These high correlations verified that initial degradation followed first-order reaction kinetics. All k_obs_, R^2^, and t_90_ values are reported in Table 1, and a typical example of linear regressions for the range of temperatures studied is shown in Fig. 3A. The general trend was that at a specified temperature, all 1 mg/mL solutions had k_obs_ values that were not statistically different from one another (p  >  0.05), and 20 mg/mL solutions all had k_obs_ values that were not statistically different from one another; however, k_obs_ values for 20 mg/mL solutions were significantly (p  <  0.05) higher than k_obs_ values for 1 mg/mL solutions. For example, at 70 °C, solutions of TMN with HNO_3_, TMN with HCl, TClHCl with HNO_3_, and TClHCl with HCl (all 1 mg/mL) had k_obs_ values of 0.043, 0.040, 0.046, and 0.047 day^−1^, respectively; conversely, the same sample types at the higher 20 mg/mL concentration had k_obs_ values of 0.43, 0.40, 0.39, and 0.38 day^−1^, respectively. Thus, all k_obs_ values of 20 mg/mL solutions were significantly higher (p  <  0.05) than the k_obs_ values of 1 mg/mL samples. Differences between k_obs_ values for each thiamine concentration were found to be larger in this study than the previous study by Voelker et al. [16]; however, the previous study did not control the initial pH, thus the difference in pH due to difference in concentration could account for the discrepancy. The k_obs_ values found in this study were consistent with those found at ambient temperatures in buffered solutions at 0.2 mg/mL TClHCl and pH 5–6, though it was noted that buffer type and concentration greatly affected these values [24]. Although there have been more reports of thiamine degradation kinetics in aqueous solutions, most are done at much higher temperatures so are therefore not compared here [31–33].

**Table 1:** Rate constants and t_90_ values for thiamine in solutions of TMN and TClHCl

pH	Vitamin salt form	Acid	Concentration (mg/mL)	Reaction kinetics	25 °C	40 °C	60 °C	70 °C	80 °C
3	TMN	HNO_3_	1	k_obs_ (day^−1^)	–	–	0.00243 ± 5e-5^C^	0.0082 ± 5e-4^B^	0.0250 ± 6e-4^C^
				R^2^	–	–	0.9894	0.9340	0.9897
				t_90_ (days)	–	–	43.3 ± 0.9^a^	12.9 ± 0.7^c^	4.2 ± 0.1^e^
			20	k_obs_ (day^−1^)	–	–	0.00251 ± 6e-5^C^	0.0061 ± 4e-4^B^	0.0207 ± 8e-4^C^
				R^2^	–	–	0.9847	0.9206	0.9710
				t_90_ (days)	–	–	42 ± 1^ab^	17 ± 1^a^	5.1 ± 0.2^cd^
		HCl	1	k_obs_ (day^−1^)	–	–	0.00253 ± 9e-5^C^	0.0084 ± 4e-4^B^	0.0253 ± 5e-4^C^
				R^2^	–	–	0.9664	0.9598	0.9920
				t_90_ (days)	–	–	42 ± 2^abc^	12.5 ± 0.5^c^	4.17 ± 0.09^e^
			20	k_obs_ (day^−1^)	–	–	0.00249 ± 7e-5^C^	0.0069 ± 4e-4^B^	0.0208 ± 3e-4^C^
				R^2^	–	–	0.9817	0.9349	0.9950
				t_90_ (days)	–	–	42 ± 1^ab^	15.2 ± 0.9^b^	5.06 ± 0.08^d^
	TClHCl	HNO_3_	1	k_obs_ (day^−1^)	–	–	0.00246 ± 5e-5^C^	0.0068 ± 4e-4^B^	0.0177 ± 6e-4^C^
				R^2^	–	–	0.9893	0.9283	0.9829
				t_90_ (days)	–	–	42.8 ± 0.9^ab^	15.4 ± 0.9^b^	6.0 ± 0.2^b^
			20	k_obs_ (day^−1^)	–	–	0.00268 ± 8e-5^C^	0.0068 ± 3e-4^B^	0.0160 ± 4e-4^C^
				R^2^	–	–	0.9783	0.9657	0.9900
				t_90_ (days)	–	–	39 ± 1^cd^	15.5 ± 0.6^b^	6.6 ± 0.2^a^
		HCl	1	k_obs_ (day^−1^)	–	–	0.00275 ± 6e-5^C^	0.0088 ± 4e-4^B^	0.0245 ± 6e-4^C^
				R^2^	–	–	0.9896	0.9584	0.9918
				t_90_ (days)	–	–	38.3 ± 0.8^d^	12.0 ± 0.5^c^	4.3 ± 0.1^e^
			20	k_obs_ (day^−1^)	–	–	0.00259 ± 9e-5^C^	0.0068 ± 3e-4^B^	0.0195 ± 6e-4^C^
				R^2^	–	–	0.9674	0.9638	0.9837
				t_90_ (days)	–	–	41 ± 1^bcd^	15.5 ± 0.6^b^	5.4 ± 0.2^c^
6	TMN	HNO_3_	1	k_obs_ (day^−1^)	0.00111 ± 4e-5^B^	0.00149 ± 6e-5^C^	0.022 ± 1e-3^C^	0.043 ± 2e-3^B^	0.122 ± 4e-3^B^
				R^2^	0.9712	0.9446	0.9330	0.9518	0.9883
				t_90_ (days)	95 ± 3^c^	70. ± 3^b^	4.7 ± 0.3^e^	2.5 ± 0.1^d^	0.86 ± 0.03^f^
			20	k_obs_ (day^−1^)	0.0058 ± 4e-4^A^	0.023 ± 2e-3^A^	0.31 ± 3e-2^A^	0.43 ± 6e-2^A^	0.48 ± 4e-2^A^
				R^2^	0.9261	0.9431	0.9312	0.8945	0.9464
				t_90_ (days)	18 ± 1^d^	4.6 ± 0.4^c^	0.34 ± 0.03^f^	0.24 ± 0.03^e^	0.22 ± 0.02^g^
		HCl	1	k_obs_ (day^−1^)	0.00067 ± 5e-5^B^	0.00133 ± 7e-5^C^	0.0196 ± 7e-4^C^	0.040 ± 1e-3^B^	0.129 ± 5e-3^B^
				R^2^	0.8943	0.9199	0.9771	0.9820	0.9803
				t_90_ (days)	160 ± 10^a^	79 ± 4^a^	5.4 ± 0.2^e^	2.63 ± 0.09^d^	0.82 ± 0.03^f^
			20	k_obs_ (day^−1^)	0.0058 ± 3e-4^A^	0.023 ± 2e-3^A^	0.29 ± 3e-2^AB^	0.40 ± 5e-2^A^	0.44 ± 6e-2^A^
				R^2^	0.9259	0.9434	0.9238	0.9051	0.8809
				t_90_ (days)	18 ± 1^d^	4.6 ± 0.4^c^	0.37 ± 0.03^f^	0.26 ± 0.03^e^	0.24 ± 0.03^g^
	TClHCl	HNO_3_	1	k_obs_ (day^−1^)	0.00080 ± 3e-5^B^	0.00129 ± 5e-5^C^	0.019 ± 1e-3^C^	0.046 ± 1e-3^B^	0.098 ± 8e-3^BC^
				R^2^	0.9616	0.9621	0.9422	0.9918	0.9244
				t_90_ (days)	131 ± 5^b^	82 ± 3^a^	5.5 ± 0.3^e^	2.28 ± 0.05^d^	1.08 ± 0.09^f^
			20	k_obs_ (day^−1^)	0.0058 ± 4e-4^A^	0.015 ± 2e-3^B^	0.26 ± 4e-2^B^	0.39 ± 5e-2^A^	0.43 ± 7e-2^A^
				R^2^	0.8974	0.8154	0.8243	0.8890	0.8289
				t_90_ (days)	18 ± 1^d^	6.8 ± 0.9^c^	0.41 ± 0.06^f^	0.27 ± 0.04^e^	0.24 ± 0.04^g^
		HCl	1	k_obs_ (day^−1^)	0.00102 ± 6e-5^B^	0.00136 ± 5e-5^C^	0.019 ± 1e-3^C^	0.047 ± 1e-3^B^	0.107 ± 3e-3^BC^
				R^2^	0.9156	0.9576	0.9334	0.9896	0.9903
				t_90_ (days)	103 ± 6^c^	77 ± 3^ab^	5.4 ± 0.3^e^	2.25 ± 0.06^d^	0.99 ± 0.02^f^
			20	k_obs_ (day^−1^)	0.0058 ± 4e-4^A^	0.016 ± 2e-3^B^	0.25 ± 4e-2^B^	0.38 ± 5e-2^A^	0.42 ± 6e-2^A^
				R^2^	0.8957	0.8263	0.8293	0.9076	0.8621
				t_90_ (days)	18 ± 1^d^	6.7 ± 0.9^c^	0.41 ± 0.06^f^	0.27 ± 0.03^e^	0.25 ± 0.04^g^

t_90_ indicates time when 90% of the initial concentration of thiamine remains

Uppercase superscript letters denote statistical significance of k_obs_ within a temperature (down columns)

Lowercase superscript letters denote statistical significance of t_90_ within a temperature (down columns)

Standard error of the slope was used for statistical calculations

Generally, a first-order reaction should have the same k value, regardless of starting concentration, and only rate should change [37], which is consistent with some thiamine degradation kinetics studies [34, 35]; however, this was not the case in the current study. Previous studies have shown concentration in solution to affect k_obs_ values, for example, in some green tea catechins [36]. This is generally attributed to the existence of multiple degradation pathways, which is known to be true of thiamine degradation. Another possible explanation for the change in k may be that the reaction order is not actually 1. Using the van’t Hoff method, the order of this reaction was calculated to be approximately 1.3. Fractional order reactions are common when degradation products participate in subsequent chemical chain reactions, which is probable in the case of thiamine degradation [30, 37]. In weakly acidic to neutral solutions (e.g., pH 6), thiamine is susceptible to hydrolysis in which the methylene bridge is broken, resulting in intact pyrimidine and thiazole moieties [18, 34, 38, 39]. The resulting intact rings are then likely to undergo subsequent reactions. If consecutive reactions are occurring, i.e. the degradation products are further reacting, the degradation reaction becomes:

$$Thiamine\to ^{{k_{1} }} degradation product 1\to ^{{k_{2} }} degradation product 2$$

in which concentration of thiamine and concentration of degradation product 1 both affect the reaction order, and k_1_ and k_2_ both contribute to k_obs_ [40]. It is also possible that intact thiamine may react with some of its degradation products, contributing an additional k value that also affects k_obs_. This consequently results in a reaction order between 1 and 2, a range which encompasses the reaction order of 1.3 in the case of this study. Since the concentration of degradation product 1 is affected by the initial concentration of thiamine, it is therefore possible that the observed k value, which incorporates both k_1_ and k_2_, and was calculated with first-order reaction equations, was affected by the initial concentration of thiamine. Concerning thiamine, the model of the participation of degradation products in consecutive degradation reactions is simplified, in which probable degradation products and subsequent consecutive reactions are much greater [18, 30]. In agreement with the proposed consecutive reaction mechanisms, it has also been suggested previously that the overall observed rate of thiamine degradation is actually a summation of a large number of separate reactions [34]. Thus, since the k_obs_ values reported in this study were presumably a function of a substantial number of k values, the variation in k_obs_ was dependent on initial thiamine concentration.

Additionally, as ionic strength increases in thiamine solutions, k values for thiamine degradation are known to significantly increase, specifically at weakly acidic or neutral pHs [34]. Since thiamine solutions in this study were prepared using salt forms of thiamine, the ionic strength of the solutions was increased as the thiamine concentration increased. It is possible that increased ionic strength in higher concentration solutions played a role in the increased k values observed in 20 mg/mL thiamine salt solutions compared to 1 mg/mL solutions in this study. Although rate constant has been reported to be independent of initial thiamine concentration in some previous studies, these systems were pH adjusted using buffers [34] or unadjusted in food systems [35], which provide additional considerations to thiamine stability.

E_a_ was calculated using the natural log of the temperature-dependent k_obs_ values for each sample type (R^2^  =  0.9465–0.9718). The Arrhenius plots used to calculate E_a_ are provided in Fig. 3B, and calculated E_a_s are reported in Table 2. In pH 6 solutions, E_a_s ranged from 18 to 21 kcal/mol, with only TMN with HCl 1 and 20 mg/mL significantly differing from one another (p  <  0.05); thus, it was concluded that all pH 6 samples underwent the same degradation pathway. These values were slightly lower than E_a_s found for dilute solutions in previous studies at similar pHs [16, 32–34, 41]; however, the calculated values in this study are still in the general range reported for thiamine degradation overall (20–30 kcal/mol) [42, 43].

**Table 2:** Calculated activation energies as a function of temperature

pH	Vitamin salt form	Acid	Concentration (mg/mL)	E_a_ (kcal/mol)	E_a_ (kJ/mol)
3	TMN	HNO_3_	1	27.2 ± 0.3^a^	114 ± 1^a^
			20	25 ± 1^ab^	103 ± 5^ab^
		HCl	1	26.9 ± 0.3^a^	113 ± 1^a^
			20	24.8 ± 0.5^ab^	104 ± 2^ab^
	TClHCl	HNO_3_	1	23.1 ± 0.4^bc^	97 ± 2^bc^
			20	20.9 ± 0.3^cd^	87 ± 1^cd^
		HCl	1	25.6 ± 0.3^ab^	107 ± 1^ab^
			20	23.6 ± 0.5^b^	99 ± 2^b^
6	TMN	HNO_3_	1	19 ± 1^de^	79 ± 5^de^
			20	18 ± 1^de^	77 ± 5^de^
		HCl	1	21 ± 1^cd^	87 ± 4^cd^
			20	18 ± 1^e^	75 ± 5^e^
	TClHCl	HNO_3_	1	20. ± 1^de^	82 ± 5^de^
			20	18 ± 1^de^	77 ± 5^de^
		HCl	1	19 ± 1^de^	80. ± 5^de^
			20	18 ± 1^de^	76 ± 5^de^

Superscript letters denote statistical significance of E_a_ (down columns)

Standard error of the slope was used for statistical calculations

### Chemical stability of thiamine in pH 3 solutions

**Fig. 4:**
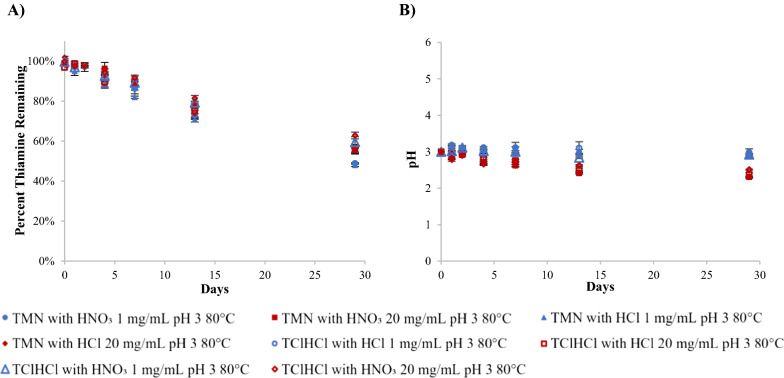
Chemical behavior of thiamine in pH 3 solutions with varying counterion (NO_3_^−^ or Cl^−^) and concentration (1 or 20 mg/mL) at 80 °C over time including: **A** degradation profiles and **B** pH profiles

**Fig. 5:**
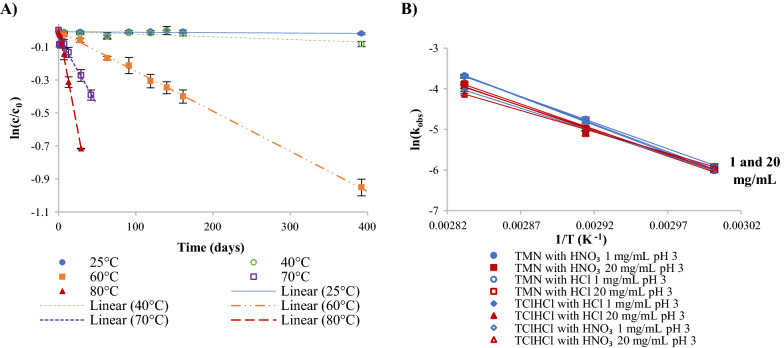
Reaction kinetics of thiamine degradation in pH 3 thiamine solutions: **A** first-order degradation regression lines of 1 mg/mL TMN solutions adjusted to pH 3 with HNO_3_ and NaOH at temperatures from 25 to 80 °C; and **B** Arrhenius plots used to calculate temperature-dependent activation energy for thiamine degradation in 1 and 20 mg/mL thiamine solutions adjusted to pH 3 with HNO_3_ or HCl and NaOH from 60 to 80 °C

To analyze thiamine stability in an acidic environment, thiamine solutions were adjusted to pH 3 and monitored for stability over time. Both temperature and molar concentration were found to significantly (p  <  0.05) affect the stability of thiamine in pH 3 solutions, with higher temperatures and higher molar concentrations causing faster degradation (Figs. 4A, 5A). Generally, percent thiamine remaining in 1 mg/mL and 20 mg/mL solutions at the same temperature and timepoint were not statistically different from one another (p  >  0.05). This can be exemplified by the percent thiamine remaining on day 91 following storage at 60 °C. Solutions of TMN with HNO_3_, TMN with HCl, TClHCl with HNO_3_, and TClHCl with HCl (all 1 mg/mL) contained 81, 80, 83, and 78% thiamine, respectively, and the same sample types at 20 mg/mL contained 82, 83, 82, and 84% thiamine, respectively. However, when comparing molar concentrations instead of percent thiamine remaining, the 20 mg/mL solutions tended to degrade faster than 1 mg/mL solutions. This is typical of a first-order reaction and is in agreement with what was found in previous studies at a similar pH [11, 16]. Thiamine was exceptionally stable over the 1-year experiment period in pH 3 solutions when stored at 25 °C or 40 °C. After 392 days of storage at these temperatures, the thiamine content in all solutions remained above 91% of the initial concentration; in most cases, there was no significant (p  <  0.05) degradation over the 392-day period. This suggests that in an acidic environment, thiamine will remain quite stable if kept below 40 °C.

An example of a typical degradation profile of thiamine at pH 3 is shown in Fig. 4A, with data for all temperatures, concentrations, and counterions provided in the Additional file 1: Tables S1, S2. The pH was also monitored over the duration of the experiment, and an example of a typical pH change over time for solutions that were initially pH 3 is shown in Fig. 4B. The pH of all samples following storage remained above 2 for the duration of the study, with most samples remaining above a pH of 2.5. The pH gradually decreased over the duration of the experiment, with 20 mg/mL solutions dropping to lower pHs than 1 mg/mL solutions. Tables including all pH data over time for all temperatures, concentrations, and counterions can be found in the Additional file 1: Tables S3, S4.

### Degradation kinetics of thiamine in pH 3 solutions

Using the van’t Hoff method, the order of the thiamine degradation reaction in pH 3 solutions was calculated to be 1, consistent with reports of thiamine degradation as a first-order reaction [11, 16]. Therefore, Eq. 1 was used to calculate the observed reaction rate constant (k_obs_) for each sample preparation of pH 3 solutions. High correlations were observed for all linear regressions of the natural log of percent thiamine remaining vs. time (R^2^  =  0.92–0.995), which, in addition to the van’t Hoff calculations, verified that initial thiamine degradation in pH 3 solutions followed first-order reaction kinetics. All k_obs_, R^2^, and t_90_ values are reported in Table 1, and a typical example of linear regressions for the range of temperatures studied is shown in Fig. 5A. Although Fig. 5A includes linear regressions for all temperatures studied, not enough thiamine degradation at 25 °C or 40 °C occurred over the duration of the 1-year experiment to allow subsequent reaction kinetics calculations from these temperatures. Thus, reaction kinetics for pH 3 solutions were only calculated for the temperatures 60, 70, and 80 °C.

At a specified temperature, 1 mg/mL and 20 mg/mL thiamine salt solutions had no k_obs_ values that were statistically different from one another (p  >  0.05). For example, at 60 °C, TMN with HNO_3_, TMN with HCl, TClHCl with HNO_3_, and TClHCl with HCl (all 1 mg/mL) had k_obs_ values of 0.00243, 0.00253, 0.00246, and 0.00275 day^−1^, respectively, and the same sample types at 20 mg/mL had k_obs_ values of 0.00251, 0.00249, 0.00268, and 0.00259 day^−1^, respectively. The k_obs_ values obtained in this study for thiamine solutions at pH 3 were similar to those reported in a previous study in solutions of the same concentrations and a similar pH range, although pH was unmodified in that study [16].

Unlike what was found in pH 6 solutions in this study, thiamine degradation in the different pH 3 solutions all had the same k value, regardless of initial thiamine concentration, which follows what is expected of a first-order reaction and is in agreement with studies by Tong et al. [35] and Windheuser and Higuchi [34]. Based on previous studies in which sensory tests were completed to compare sensory properties of thiamine degraded in acidic vs. close to neutral solutions, we know that the degradation pathway differs between pH 3 and pH 6 solutions [16]. The difference in degradation pathway was presumably due to hydrolysis of the pyrimidine and thiazole moieties of thiamine not being the major degradation pathway in the pH 3 environment, as has been suggested previously [34]. Thus, the first thiamine degradation step in pH 3 solutions was presumably the rate-determining step. Consequently, k values of the consecutive reactions of the degradation products did not significantly affect the k_obs_ values, resulting in k_obs_ values at pH 3 that were not statistically different from one another. Additionally, it has been reported that although k values of thiamine degradation are highly dependent on ionic strength in pH 6 solutions, k values in acidic pHs are independent of ionic strength [34]; thus, the difference in ionic strength resulting from the different concentrations of thiamine salt forms did not play a role in k values of thiamine degradation in pH 3 solutions as was found in pH 6 solutions.

E_a_ was calculated using the natural log of the temperature-dependent k_obs_ values for each pH 3 sample type (R^2^  =  0.9861–0.9990). The Arrhenius plots used to calculate E_a_ are shown in Fig. 5B, and calculated E_a_ values are reported in Table 2. In thiamine solutions at pH 3, E_a_ values ranged from 21 to 27 kcal/mol. There were some significant differences between E_a_ values (p  <  0.05); however, the small range of E_a_ values indicates that all pH 3 sample preparations likely underwent the same degradation pathway. Although reports of reaction kinetics of thiamine degradation at approximately pH 3 are limited, the E_a_ values found in this study are in ranges reported previously (20–30 kcal/mol), albeit at different pHs and complexity of the systems [20, 32, 33, 41–43]. In similar pH systems, including without the use of buffer salts, the E_a_ values in this study are also in accordance with what has previously been reported [16, 34].

### Comparison of pH- and counterion-dependent thiamine stability

**Fig. 6:**
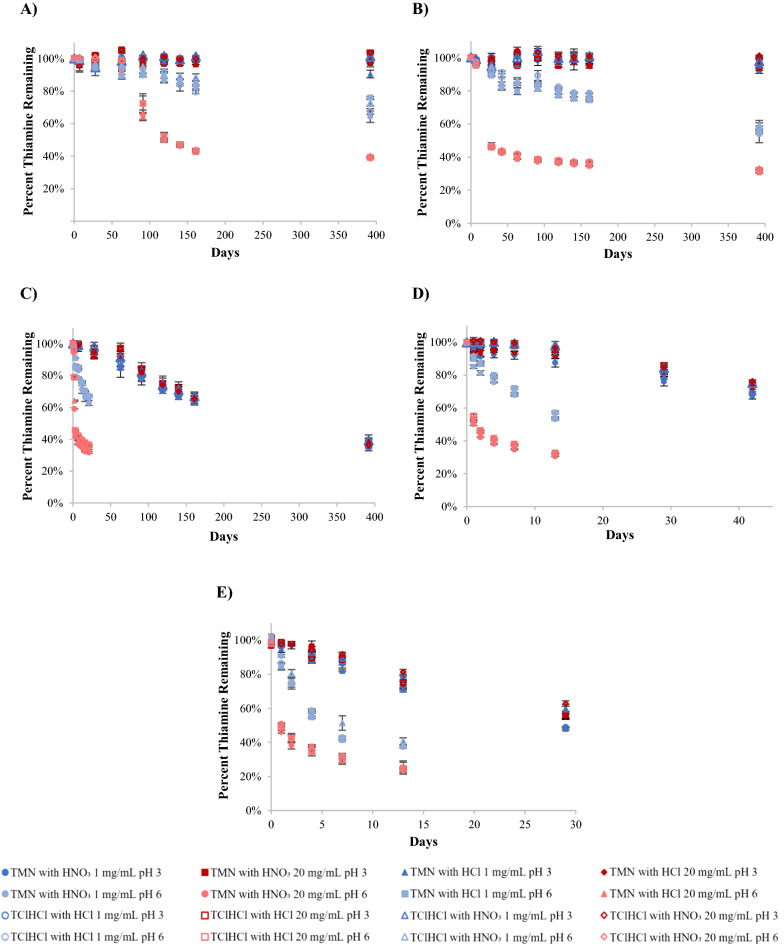
Comparison of chemical stability over time of thiamine in pH 3 (darker colored data points) vs. pH 6 (lighter colored data points) solutions at 1 and 20 mg/mL concentrations stored at **A** 25 °C, **B** 40 °C, **C** 60 °C, **D** 70 °C, **E** 80 °C

The stability of thiamine in solution was significantly higher in pH 3 solutions than in pH 6 solutions (Fig. 6), consistent with reports at many temperatures (from ambient to those found during processing) as well as in a variety of matrices (buffer systems and food products), commonly attributed to different thiamine degradation mechanisms at different pHs [19, 24, 31, 34]. Although all pH 3 and 1 mg/mL pH 6 solutions tended to have k_obs_ values that were not statistically different from one another (p  >  0.05), the 20 mg/mL pH 6 solutions had k_obs_ values a factor of 10 greater than k_obs_ values for pH 3 solutions in all cases (Table 1), indicating increased stability in all pH 3 solutions. In addition, the E_a_s of thiamine degradation in pH 6 solutions tended to be significantly (p  <  0.05) lower than the E_a_s in pH 3 solutions (Table 2). The k_obs_ values, E_a_s, and the percent remaining graphs over time at each temperature (Fig. 6) verify previous reports that thiamine is more stable in acidic environments [16, 30]. Additionally, since the E_a_ of thiamine degradation was higher in pH 3 solutions than in pH 6 solutions, it was concluded that the thiamine degradation pathway in the two pH environments differed, as was also suggested by sensory data in our previous study [16]. However, as expected, both concentrations had the same E_a_ at their respective pHs, indicating that thiamine concentration does not affect degradation pathway.

Since the presence of salts is known to affect thiamine stability, we prepared thiamine solutions adjusted to both pHs (3 and 6) using both salt forms of thiamine (TMN and TClHCl) adjusted with acids (HNO_3_ or HCl) that would either isolate one counterion in solution or introduce both salt form counterions (NO_3_^−^ or Cl^−^) in solution to determine if counterion had an effect on stability. The k_obs_ and E_a_ values (Tables 1, 2) as well as percent thiamine remaining over time (Fig. 6) illustrated that regardless of the counterion(s) present in solution, the thiamine degradation reaction proceeded in the same manner. Additionally, all E_a_s calculated in this study were similar to those reported at the same thiamine salt concentrations (1 and 20 mg/mL) and pHs without pH adjustment [16]; thus, it was determined that the presence of Cl^−^ or NO_3_^−^ (as the pH adjustment mechanism) did not trigger a change in the thiamine degradation pathway in this study. Therefore, pH and concentration were considered to be the sole factors contributing to thiamine degradation kinetics in this study.

The extent of the effect of pH on reaction kinetics of thiamine degradation can be quantified by graphing the log of k_obs_ as a function of pH, in which a resulting slope of 1 would indicate an ideal acid–base catalyzed reaction. In studies by Pachapurkar and Bell [24] and Windheuser and Higuchi [34], it was found that although there is a high correlation between log of the rate constant and pH (in the pH range 4–7), the slope of the plot indicates that the effects of pH are more complex than the ideal acid–base catalyzed reaction. They also found that the sensitivity of thiamine degradation to pH is dependent on the type of buffer, in which thiamine in a phosphate buffer system is more sensitive to pH than in a citrate buffer system, and correlation between reaction rate constant and pH is lower in citrate buffers than in phosphate buffers, presumably due to the stronger ability of phosphate to deprotonate thiamine than citrate [24]. Although the current study used only two pHs (3 and 6), when the data were plotted as log(k_obs_) vs. pH, the slopes for 1 mg/mL and 20 mg/mL thiamine solutions were 0.23–0.30 and 0.45–0.68, respectively, dependent on temperature. As in the previous studies, these slopes indicated that the thiamine degradation reaction is less dependent on pH than an ideal acid–base catalyzed reaction. Additionally, these values suggest that the stability of thiamine in 20 mg/mL solutions is more influenced by a change in pH than in 1 mg/mL solutions. Overall, it was concluded that a change in solution pH caused a change in the rate of thiamine degradation (and rate constant), the E_a_ required for degradation, the degradation pathway, and therefore, the resulting degradation products, which has been shown in previous studies to have a significant sensory impact due to sulfur containing degradation products [16, 17, 44].

### Potential impact on food formulation

Although this study investigated thiamine stability in simple aqueous solutions at higher thiamine concentrations than are often found in food products, understanding the fundamental reaction mechanisms of thiamine degradation can be used to predict the responses in a variety of food formulations and vitamin supplements. Thiamine has been reported to be more stable in food systems than in buffer systems, and thiamine degradation has been shown to both deviate from and to follow first-order reaction kinetics in food products due to interactions with food ingredients [31, 41, 45]. Regardless of order of reaction, certain components in food are known to affect the stability of thiamine. For example, α- and β-amino acids and their derivatives, proteins, and starch have been shown to stabilize thiamine in foods, attributed to changes in molecular mobility and/or chemical interactions between functional groups on the ingredients and thiamine, but salts and sulfites are known to destabilize thiamine, also due to intermolecular interactions [13, 14, 41, 46].

Many foods, including fruit juices, sports drinks, and energy drinks, offer the protective effect of an acidic environment on thiamine. Many other thiamine-containing foods, such as eggs, milk, infant formulas, and other dairy-based nutritional beverages, have a close to neutral or even slightly alkaline pH, which was shown to significantly decrease thiamine stability. The pH- and concentration-based reaction kinetics and analytical methods in this study were used to monitor thiamine stability in a bread dough system, in which it was shown that thiamine was more stable in the bread dough system than in an aqueous solution of a similar pH due to interactions with bread dough ingredients, including starch and gluten [47]. This method can also be used to predict thiamine stability in a variety of other food products. While thiamine will behave differently in most distinctive food matrices, as demonstrated in the bread dough system, the degradation kinetics reported in this study provide a basis for this understanding using the fundamental stability of thiamine. Analyzing thiamine behavior in model food-formulations using guidance from the conclusions of this study may also extend the implications of this study to include an understanding of thiamine in specific food formulations.

## Conclusion

Degradation kinetics of thiamine in solution were shown to be highly dependent on pH, concentration, and storage temperature, but the degradation was not affected by counterion present (NO_3_^−^ vs. Cl^−^) in the aqueous solutions. Thiamine was significantly (p  <  0.05) more stable in pH 3 solutions than in pH 6 solutions. Additionally, differences in E_a_ values found for thiamine degradation at the two pHs indicated a difference in degradation reaction pathway between the two solution environments. All thiamine degradation was shown to follow first-order reaction kinetics; however, thiamine at pH 6 degraded via a pseudo first-order reaction (reaction order 1.3), whereas thiamine at pH 3 degraded via an ideal first-order reaction. The initial thiamine concentration was found to have a significant effect on thiamine stability in pH 6 solutions, with higher concentrations increasing k_obs_, but k_obs_ values of thiamine in pH 3 solutions were not dependent on initial concentration. This difference was due to the difference in thiamine degradation pathway at different pHs as well as differences of response to ionic strength: ionic strength affects k values in pH 6 solutions but not in pH 3 solutions. This study developed long term thiamine stability studies focusing on the effect of pH and thiamine concentration without the use of buffers. The fundamental understanding of the response of thiamine to a variety of matrices and temperatures can be used to improve thiamine delivery in food products.

## Supplementary Information

**Additional file 1: Table S1.** Percent TMN remaining after storage at the specified conditions over time: A) 25 °C, B) 40 °C, C) 60 °C, D) 70 °C, and E) 80 °C. **Table S2.** Percent TClHCl remaining after storage at the specified conditions over time: A) 25 °C, B) 40 °C, C) 60 °C, D) 70 °C, and E) 80 °C. **Table S3.** pH of TMN solutions after storage at the specified conditions over time: A) 25 °C, B) 40 °C, C) 60 °C, D) 70 °C, and E) 80 °C. **Table S4.** pH of TClHCl solutions after storage at the specified conditions over time: A) 25 °C, B) 40 °C, C) 60 °C, D) 70 °C, and E) 80 °C.

## Data Availability

The datasets supporting the conclusions of this article are included within the article and its additional files.

## Abbreviations

TMN: Thiamine mononitrate
TClHCl: Thiamine chloride hydrochloride
HPLC: High-performance liquid chromatography
Cl^−^: Chloride ion
NO_3_^−^: Nitrate ion
HCl: Hydrochloric acid
HNO_3_: Nitric acid
NaOH: Sodium hydroxide
E_a_: Activation energy
k_obs_: Observed reaction rate constant
