# Supplementary material for: Effect of pH and concentration on the chemical stability and reaction kinetics of thiamine mononitrate and thiamine chloride hydrochloride in solution
Source: BMC Chem. 2021 Aug 12;15(1):47. doi: 10.1186/s13065-021-00773-y (PMC8362232; doi:10.1186/s13065-021-00773-y)
Supplement: Supplementary file 1 — Additional file 1: Table S1. Percent TMN remaining after storage at the specified conditions over time: A) 25 °C, B) 40 °C, C) 60 °C, D) 70 °C, and E) 80 °C. Table S2. Percent TClHCl remaining after storage at the specified conditions over time: A) 25 °C, B) 40 °C, C) 60 °C, D) 70 °C, and E) 80 °C. Table S3. pH of TMN solutions after storage at the specified conditions over time: A) 25 °C, B) 40 °C, C) 60 °C, D) 70 °C, and E) 80 °C. Table S4. pH of TClHCl solutions after storage at the specified conditions over time: A) 25 °C, B) 40 °C, C) 60 °C, D) 70 °C, and E) 80 °C. [file 13065_2021_773_MOESM1_ESM.docx]

Additional Files

**Additional Table S1.** Percent TMN remaining after storage at the specified conditions over time: **A)** 25ºC, **B)** 40ºC, **C)** 60ºC, **D)** 70ºC, and **E)** 80ºC. Uppercase superscript letters on values for each sample type denote statistical significance within that sample type (down columns). Lowercase superscript letters denote statistical significance between sample types for each day (across rows).

**A)**

| **TMN 25ºC**  **Percent Vitamin Remaining** | | | | | | | | |
| --- | --- | --- | --- | --- | --- | --- | --- | --- |
| **Days** | **pH 3** | | | | **pH 6** | | | |
|  | **TMN with HNO_3_** | | **TMN with HCl** | | **TMN with HNO_3_** | | **TMN with HCl** | |
|  | **1 mg/mL** | **20 mg/mL** | **1 mg/mL** | **20 mg/mL** | **1 mg/mL** | **20 mg/mL** | **1 mg/mL** | **20 mg/mL** |
| 0 | 100.1 ± 0.3% ^Aa^ | 100. ± 2% ^Aa^ | 100.1 ± 0.3% ^Aa^ | 100.0 ± 0.7% ^Aa^ | 100.1 ± 0.8% ^Aa^ | 100. ± 2% ^Aa^ | 100.0 ± 0.4% ^Aa^ | 100.1 ± 0.5% ^Aa^ |
| 7 | 99.1 ± 0.6% ^ABabc^ | 98.7 ± 0.4% ^ABabc^ | 98 ± 1% ^Ac^ | 100.3 ± 0.4% ^Aa^ | 98.5 ± 0.4% ^Abc^ | 99.3 ± 0.1% ^Aabc^ | 98.1 ± 0.2% ^ABc^ | 99.9 ± 0.4% ^Aab^ |
| 28 | 99.1 ± 0.6% ^ABa^ | 98.0 ± 0.3% ^BCa^ | 98.2 ± 0.4% ^Aa^ | 99.2 ± 0.2% ^Aa^ | 96.5 ± 0.6% ^Aa^ | 99 ± 4% ^Aa^ | 95.3 ± 0.6% ^Ba^ | 100. ± 3% ^Aa^ |
| 63 | 97 ± 2% ^Bab^ | 97.6 ± 0.9% ^BCab^ | 96 ± 3% ^Aab^ | 99.6 ± 0.2% ^Aa^ | 90. ± 3% ^Bc^ | 93 ± 1% ^Bbc^ | 89 ± 1% ^CDc^ | 93.1 ± 0.7% ^Bbc^ |
| 91 | 99.3 ± 0.7% ^Aa^ | 96.5 ± 0.9% ^Ca^ | 99.7 ± 0.7% ^Aa^ | 99 ± 1% ^Aa^ | 89.6 ± 0.9% ^Bb^ | 65 ± 3% ^Cc^ | 90.0 ± 0.9% ^Cb^ | 64 ± 2% ^Cc^ |
| 119 | 99.3 ± 0.7% ^Aa^ | 96.8 ± 0.4% ^BCa^ | 98.3 ± 0.4% ^Aa^ | 100. ± 1% ^Aa^ | 87 ± 1% ^BCb^ | 50.1 ± 0.1% ^Dc^ | 88 ± 3% ^CDb^ | 50. ± 1% ^Dc^ |
| 140 | 99.5 ± 0.7% ^Aa^ | 96.6 ± 0.7% ^BCa^ | 99.3 ± 0.9% ^Aa^ | 100. ± 1% ^Aa^ | 85 ± 2% ^BCb^ | 46.1 ± 0.5% ^DEc^ | 86 ± 1% ^DEb^ | 46.5 ± 0.6% ^DEc^ |
| 161 | 99 ± 1% ^ABa^ | 96.4 ± 0.1% ^Cb^ | 99 ± 1% ^Aab^ | 98.6 ± 0.9% ^Aab^ | 83 ± 1% ^Cc^ | 43.8 ± 0.2% ^Ed^ | 85 ± 1% ^Ec^ | 44.3 ± 0.4% ^Ed^ |
| 392 | 98.0 ± 0.5% ^ABa^ | 97.29 ± 0.06% ^BCa^ | 91 ± 2% ^Bb^ | 99.6 ± 0.2% ^Aa^ | 64 ± 4% ^Dd^ | 38.48 ± 0.06% ^Fe^ | 76.1 ± 0.7% ^Fc^ | 39.1 ± 0.2% ^Fe^ |

**B)**

| **TMN 40ºC**  **Percent Vitamin Remaining** | | | | | | | | |
| --- | --- | --- | --- | --- | --- | --- | --- | --- |
| **Days** | **pH 3** | | | | **pH 6** | | | |
|  | **TMN with HNO_3_** | | **TMN with HCl** | | **TMN with HNO_3_** | | **TMN with HCl** | |
|  | **1 mg/mL** | **20 mg/mL** | **1 mg/mL** | **20 mg/mL** | **1 mg/mL** | **20 mg/mL** | **1 mg/mL** | **20 mg/mL** |
| 0 | 100.1 ± 0.3% ^Aa^ | 100. ± 2% ^Aa^ | 100.1 ± 0.3% ^ABCa^ | 100.0 ± 0.7% ^Aa^ | 100.1 ± 0.8% ^Aa^ | 100. ± 2% ^Aa^ | 100.0 ± 0.4% ^Aa^ | 100.1 ± 0.5% ^Aa^ |
| 7 | 98.7 ± 0.6% ^ABa^ | 97.2 ± 0.5% ^Abc^ | 98.6 ± 0.5% ^ABCa^ | 98.4 ± 0.2% ^Aab^ | 96.8 ± 0.8% ^ABc^ | 96.1 ± 0.2% ^Bcd^ | 95.3 ± 0.2% ^Bd^ | 96.5 ± 0.2% ^Bcd^ |
| 28 | 97.9 ± 0.2% ^ABa^ | 95.6 ± 0.4% ^Aab^ | 97.4 ± 0.1% ^ABCa^ | 99 ± 4% ^Aa^ | 91.5 ± 0.2% ^Bbc^ | 47 ± 2% ^Cd^ | 89.1 ± 0.4% ^Cc^ | 47.3 ± 0.1% ^Cd^ |
| 42 | - | - | - | - | 84 ± 4% ^Ca^ | 42.3 ± 0.1% ^Db^ | 82 ± 1% ^Da^ | 42.6 ± 0.5% ^Db^ |
| 63 | 96.3 ± 0.9% ^Ba^ | 95.0 ± 0.5% ^Aa^ | 96 ± 1% ^BCa^ | 96.9 ± 0.7% ^Aa^ | 84.3 ± 0.9% ^Cb^ | 38.9 ± 0.1% ^Ed^ | 80. ± 2% ^DEc^ | 39.00 ± 0.02 ^Ed^ |
| 91 | 98.4 ± 0.4% ^ABa^ | 99 ± 4% ^Aa^ | 103 ± 3% ^Aa^ | 99 ± 1% ^Aa^ | 83.4 ± 0.6% ^CDb^ | 37.1 ± 0.3% ^EFc^ | 81 ± 1% ^Db^ | 38.1 ± 0.5% ^Ec^ |
| 119 | 98.2 ± 0.7% ^ABa^ | 95 ± 1% ^Ab^ | 97 ± 2% ^ABCab^ | 97 ± 1% ^Aab^ | 79.3 ± 0.9% ^CDEc^ | 36.1 ± 0.3% ^FGd^ | 77.0 ± 0.9% ^EFc^ | 36.8 ± 0.3% ^Fd^ |
| 140 | 100. ± 2% ^Aa^ | 98 ± 5% ^Aa^ | 101 ± 4% ^ABa^ | 96.9 ± 0.9% ^Aa^ | 78 ± 1% ^DEb^ | 35.5 ± 0.1% ^FGc^ | 75 ± 1% ^Fb^ | 35.9 ± 0.3% ^Fc^ |
| 161 | 98 ± 1% ^ABab^ | 95 ± 1% ^Ab^ | 99 ± 3% ^ABCa^ | 95.9 ± 0.7% ^Aab^ | 75.5 ± 0.9% ^Ec^ | 34.6 ± 0.2% ^Gd^ | 74.2 ± 0.8% ^Fc^ | 34.8 ± 0.2% ^Gd^ |
| 392 | 92 ± 2% ^Ca^ | 93.8 ± 0.3% ^Aa^ | 95 ± 2% ^Ca^ | 96 ± 4% ^Aa^ | 54 ± 5% ^Fb^ | 31.2 ± 0.8% ^Hc^ | 56 ± 1% ^Gb^ | 30.73 ± 0.02% ^Hc^ |

**C)**

| **TMN 60ºC**  **Percent Vitamin Remaining** | | | | | | | | |
| --- | --- | --- | --- | --- | --- | --- | --- | --- |
| **Days** | **pH 3** | | | | **pH 6** | | | |
|  | **TMN with HNO_3_** | | **TMN with HCl** | | **TMN with HNO_3_** | | **TMN with HCl** | |
|  | **1 mg/mL** | **20 mg/mL** | **1 mg/mL** | **20 mg/mL** | **1 mg/mL** | **20 mg/mL** | **1 mg/mL** | **20 mg/mL** |
| 0 | 100.1 ± 0.3% ^Aa^ | 100. ± 2% ^Aa^ | 100.1 ± 0.3% ^Aa^ | 100.0 ± 0.7% ^Aa^ | 99.9 ± 0.4% ^Aa^ | 100. ± 1% ^Aa^ | 99.9 ± 0.6% ^Aa^ | 100.1 ± 0.7% ^Aa^ |
| 1 | - | - | - | - | 98.2 ± 0.4% ^Aa^ | 94.4 ± 0.1% ^Bc^ | 98.1 ± 0.2% ^Ba^ | 95.2 ± 0.1% ^Bb^ |
| 2 | - | - | - | - | - | 59.2 ± 0.1% ^Cb^ | - | 64.2 ± 0.5% ^Ca^ |
| 3 | - | - | - | - | 85.1 ± 0.5% ^Bb^ | 41.5 ± 0.2% ^Dd^ | 90.9 ± 0.1% ^Ca^ | 43.9 ± 0.2% ^Dc^ |
| 7 | 97.5 ± 0.8% ^Aa^ | 98 ± 3% ^ABa^ | 97.3 ± 0.9% ^ABa^ | 97.9 ± 0.3% ^ABa^ | 78.6 ± 0.4% ^Cc^ | 36.9 ± 0.3% ^Ed^ | 85 ± 1% ^Db^ | 39.3 ± 0.2% ^Ed^ |
| 12 | - | - | - | - | 71 ± 2% ^Db^ | 34.9 ± 0.1% ^Fc^ | 75.2 ± 0.2% ^Ea^ | 36.83 ± 0.08% ^Fc^ |
| 16 | - | - | - | - | 66 ± 2% ^Eb^ | 32.3 ± 0.4% ^Gc^ | 70.5 ± 0.2% ^Fa^ | 34.9 ± 0.8% ^Gc^ |
| 21 | - | - | - | - | 63 ± 2% ^Eb^ | 31.7 ± 0.5% ^Gd^ | 67.2 ± 0.6% ^Ga^ | 34.2 ± 0.6% ^Gc^ |
| 28 | 94 ± 1% ^Aa^ | 92 ± 1% ^Ba^ | 94 ± 3% ^ABa^ | 95 ± 1% ^ABa^ | - | - | - | - |
| 63 | 84.5 ± 0.9% ^Ba^ | 92 ± 3% ^Ba^ | 87 ± 8% ^BCa^ | 94 ± 2% ^Ba^ | - | - | - | - |
| 91 | 81 ± 4% ^Ba^ | 82 ± 3% ^Ca^ | 80. ± 6% ^CDa^ | 83 ± 3% ^Ca^ | - | - | - | - |
| 119 | 74 ± 3% ^Ca^ | 75 ± 2% ^Da^ | 70.6 ± 0.2% ^DEa^ | 75 ± 4% ^Da^ | - | - | - | - |
| 140 | 71 ± 3% ^CDab^ | 72 ± 2% ^DEab^ | 67.2 ± 0.1% ^Eb^ | 72 ± 2% ^DEa^ | - | - | - | - |
| 161 | 67 ± 3% ^Da^ | 66.2 ± 0.9% ^Ea^ | 65 ± 2% ^Ea^ | 67 ± 2% ^Ea^ | - | - | - | - |
| 392 | 39 ± 2% ^Ea^ | 38 ± 2% ^Fa^ | 38 ± 5% ^Fa^ | 39 ± 2% ^Fa^ | - | - | - | - |

**D)**

| **TMN 70ºC**  **Percent Vitamin Remaining** | | | | | | | | |
| --- | --- | --- | --- | --- | --- | --- | --- | --- |
| **Days** | **pH 3** | | | | **pH 6** | | | |
|  | **TMN with HNO_3_** | | **TMN with HCl** | | **TMN with HNO_3_** | | **TMN with HCl** | |
|  | **1 mg/mL** | **20 mg/mL** | **1 mg/mL** | **20 mg/mL** | **1 mg/mL** | **20 mg/mL** | **1 mg/mL** | **20 mg/mL** |
| 0 | 100.1 ± 0.1% ^Aa^ | 100. ± 1% ^Aa^ | 100.1 ± 0.3% ^Aa^ | 99.9 ± 0.5% ^Aa^ | 99.9 ± 0.3% ^Aa^ | 100. ± 1% ^Aa^ | 99.9 ± 0.6% ^Aa^ | 100.1 ± 0.7% ^Aa^ |
| 1 | 91.9 ± 0.7% ^Bcd^ | 95.0 ± 0.7% ^BCab^ | 97 ± 2% ^Aa^ | 93.65 ± 0.08% ^Bbc^ | 85 ± 1% ^Be^ | 50. ± 1% ^Bg^ | 89.8 ± 0.7% ^Bd^ | 53.5 ± 0.9% ^Bf^ |
| 2 | 91.2 ± 0.4% ^Bc^ | 95.6 ± 0.5% ^BCab^ | 97.8 ± 0.9% ^Aa^ | 93 ± 2% ^Bbc^ | 81 ± 1% ^Ce^ | 42.19 ± 0.02% ^Cf^ | 87 ± 1% ^Cd^ | 44.8 ± 0.2% ^Cf^ |
| 4 | 93 ± 2% ^Bc^ | 95.9 ± 0.3% ^BCb^ | 100. ± 2% ^Aa^ | 94.3 ± 0.4% ^Bbc^ | 75.6 ± 0.4% ^De^ | 38.2 ± 0.5% ^Df^ | 80.2 ± 0.2% ^Dd^ | 40.3 ± 0.2% ^Df^ |
| 7 | 92 ± 3% ^Bb^ | 97.4 ± 0.3% ^ABa^ | 97.9 ± 0.1% ^Aa^ | 93.4 ± 0.7% ^Bb^ | 67.8 ± 0.7% ^Ed^ | 34.8 ± 0.3% ^Ee^ | 72.2 ± 0.7% ^Ec^ | 37.3 ± 0.3% ^Ee^ |
| 13 | 88 ± 3% ^Bb^ | 94.6 ± 0.7% ^Ca^ | 93.0 ± 0.5% ^Ba^ | 91 ± 1% ^Ba^ | 53.6 ± 0.9% ^Fd^ | 30.6 ± 0.1% ^Fe^ | 57.2 ± 0.7% ^Fc^ | 32.3 ± 0.5% ^Fe^ |
| 29 | 76 ± 3% ^Cc^ | 84.2 ± 0.8% ^Da^ | 79.8 ± 0.5% ^Cbc^ | 80.6 ± 0.3% ^Cab^ | - | - | - | - |
| 42 | 68 ± 2% ^Db^ | 75 ± 2% ^Ea^ | 70. ± 2% ^Dab^ | 71 ± 3% ^Dab^ | - | - | - | - |

**E)**

| **TMN 80ºC**  **Percent Vitamin Remaining** | | | | | | | | |
| --- | --- | --- | --- | --- | --- | --- | --- | --- |
| **Days** | **pH 3** | | | | **pH 6** | | | |
|  | **TMN with HNO_3_** | | **TMN with HCl** | | **TMN with HNO_3_** | | **TMN with HCl** | |
|  | **1 mg/mL** | **20 mg/mL** | **1 mg/mL** | **20 mg/mL** | **1 mg/mL** | **20 mg/mL** | **1 mg/mL** | **20 mg/mL** |
| 0 | 99.0 ± 0.5% ^Ab^ | 99 ± 1% ^Ab^ | 100.1 ± 0.6% ^Ab^ | 101.9 ± 0.5% ^Aa^ | 98.3 ± 0.6% ^Ab^ | 98.9 ± 0.1% ^Ab^ | 102.5 ± 0.6% ^Aa^ | 102.5 ± 0.2% ^Aa^ |
| 1 | 98 ± 2% ^Aa^ | 98 ± 1% ^Aa^ | 95 ± 2% ^Ba^ | 97.9 ± 0.6% ^Ba^ | 90.8 ± 0.7% ^Bb^ | 51.0 ± 0.5% ^Bd^ | 85 ± 2% ^Bc^ | 50. ± 1% ^Bd^ |
| 2 | 97 ± 2% ^Aa^ | 97.8 ± 0.9% ^Aa^ | 97.7 ± 0.2% ^ABa^ | 97.1 ± 0.6% ^Ba^ | 74 ± 3% ^Cb^ | 38 ± 2% ^Cc^ | 75 ± 3% ^Cb^ | 42 ± 3% ^Cc^ |
| 4 | 93 ± 3% ^Aa^ | 96 ± 3% ^Aa^ | 88 ± 2% ^Cb^ | 93.1 ± 0.4% ^Cab^ | 58.9 ± 0.8% ^Dc^ | 37.7 ± 0.3% ^Cd^ | 54.7 ± 0.6% ^Dc^ | 37 ± 2% ^CDd^ |
| 7 | 87 ± 3% ^Ba^ | 90. ± 3% ^Ba^ | 81.9 ± 0.5% ^Db^ | 89 ± 1% ^Da^ | 42.5 ± 0.6% ^Ec^ | 32.17 ± 0.04% ^Dd^ | 41.4 ± 0.5% ^Ec^ | 32 ± 2% ^Dd^ |
| 13 | 73 ± 2% ^Cab^ | 74 ± 3% ^Cab^ | 71 ± 1% ^Eb^ | 78 ± 2% ^Ea^ | 37.3 ± 0.7% ^Fc^ | 24.9 ± 0.3% ^Ed^ | 25.2 ± 0.4% ^Fd^ | 26 ± 3% ^Ed^ |
| 29 | 48.8 ± 0.1% ^Db^ | 56 ± 2% ^Da^ | 47.9 ± 0.7% ^Fb^ | 55 ± 1% ^Fa^ | - | - | - | - |

**Additional Table S2.** Percent TClHCl remaining after storage at the specified conditions over time: **A)** 25ºC, **B)** 40ºC, **C)** 60ºC, **D)** 70ºC, and **E)** 80ºC. Uppercase superscript letters on values for each sample type denote statistical significance within that sample type (down columns). Lowercase superscript letters denote statistical significance between sample types for each day (across rows).

**A)**

| **TClHCl 25ºC**  **Percent Vitamin Remaining** | | | | | | | | |
| --- | --- | --- | --- | --- | --- | --- | --- | --- |
| **Days** | **pH 3** | | | | **pH 6** | | | |
|  | **TClHCl with HCl** | | **TClHCl with HNO_3_** | | **TClHCl with HCl** | | **TClHCl with HNO_3_** | |
|  | **1 mg/mL** | **20 mg/mL** | **1 mg/mL** | **20 mg/mL** | **1 mg/mL** | **20 mg/mL** | **1 mg/mL** | **20 mg/mL** |
| 0 | 100.0 ± 0.4% ^Aa^ | 100.1 ± 0.6% ^BCa^ | 100.1 ± 0.3% ^Aa^ | 100.0 ± 0.2% ^Ca^ | 100.0 ± 0.4% ^Aa^ | 100.1 ± 0.9% ^Aa^ | 100.1 ± 0.5% ^Aa^ | 100.1 ± 0.4% ^Aa^ |
| 7 | 99.0 ± 0.2% ^Aab^ | 96 ± 3% ^Dab^ | 99.1 ± 0.5% ^Aab^ | 95 ± 4% ^Db^ | 99.1 ± 0.2% ^Aab^ | 99.0 ± 0.4% ^Aab^ | 99.4 ± 0.5% ^ABab^ | 101.0 ± 0.2% ^Aa^ |
| 28 | 99.9 ± 0.1% ^Aab^ | 102.1 ± 0.2% ^ABCa^ | 95 ± 1% ^Bb^ | 98.3 ± 0.1% ^CDab^ | 94 ± 5% ^ABb^ | 99.8 ± 0.2% ^Aab^ | 96 ± 2% ^ABCab^ | 101 ± 2% ^Aa^ |
| 63 | 101 ± 2% ^Aab^ | 105.5 ± 0.6% ^Aa^ | 99 ± 2% ^ABbc^ | 105.4 ± 0.6% ^Aa^ | 94.0 ± 0.8% ^ABc^ | 99 ± 1% ^Abc^ | 96 ± 2% ^ABCbc^ | 98 ± 5% ^Abc^ |
| 91 | 100.0 ± 0.5% ^Aab^ | 100.1 ± 0.5% ^BCab^ | 101.9 ± 0.3% ^Aa^ | 100.1 ± 0.5% ^BCab^ | 93 ± 2% ^ABb^ | 72 ± 6% ^Bc^ | 95.2 ± 0.8% ^BCab^ | 72 ± 5% ^Bc^ |
| 119 | 100.9 ± 0.1% ^Aa^ | 101.2 ± 0.7% ^BCa^ | 101.7 ± 0.5% ^Aa^ | 101.1 ± 0.2% ^BCa^ | 89 ± 4% ^BCb^ | 52 ± 2% ^Cc^ | 93 ± 1% ^CDb^ | 52 ± 3% ^Cc^ |
| 140 | 99.6 ± 0.2% ^Aa^ | 99.9 ± 0.5% ^Ca^ | 99.7 ± 0.3% ^Aa^ | 99.4 ± 0.3% ^Ca^ | 84 ± 4% ^CDc^ | 46.9 ± 0.9% ^CDd^ | 89 ± 2% ^Db^ | 47 ± 2% ^CDd^ |
| 161 | 101.1 ± 0.3% ^Aa^ | 100.1 ± 0.4% ^BCa^ | 101.4 ± 0.4% ^Aa^ | 100.1 ± 0.1% ^BCa^ | 79 ± 1% ^Dc^ | 42.6 ± 0.6% ^DEd^ | 89 ± 2% ^Db^ | 43.8 ± 0.8% ^Dd^ |
| 392 | 100. ± 5% ^Aa^ | 103.5 ± 0.1% ^ABa^ | 101 ± 4% ^Aa^ | 103.8 ± 0.1% ^ABa^ | 68 ± 2% ^Eb^ | 39.1 ± 0.1% ^Ec^ | 73 ± 2% ^Eb^ | 40.0 ± 0.2% ^Dc^ |

**B)**

| **TClHCl 40ºC**  **Percent Vitamin Remaining** | | | | | | | | |
| --- | --- | --- | --- | --- | --- | --- | --- | --- |
| **Days** | **pH 3** | | | | **pH 6** | | | |
|  | **TClHCl with HCl** | | **TClHCl with HNO_3_** | | **TClHCl with HCl** | | **TClHCl with HNO_3_** | |
|  | **1 mg/mL** | **20 mg/mL** | **1 mg/mL** | **20 mg/mL** | **1 mg/mL** | **20 mg/mL** | **1 mg/mL** | **20 mg/mL** |
| 0 | 100.0 ± 0.4% ^ABa^ | 100.1 ± 0.6% ^ABa^ | 100.1 ± 0.3% ^ABCa^ | 100.0 ± 0.2% ^ABa^ | 100.0 ± 0.4% ^Aa^ | 100.1 ± 0.9% ^Aa^ | 100.1 ± 0.5% ^Aa^ | 100.1 ± 0.4% ^Aa^ |
| 7 | 98.6 ± 0.1% ^ABab^ | 98.43 ± 0.03% ^Bbc^ | 99.1 ± 0.2% ^ABCa^ | 98.0 ± 0.2% ^Bcd^ | 97.5 ± 0.1% ^Ad^ | 95.3 ± 0.4% ^Bf^ | 97.6 ± 0.2% ^ABd^ | 96.8 ± 0.3% ^Be^ |
| 28 | 100.5 ± 0.3% ^ABa^ | 97.8 ± 0.3% ^Bab^ | 95 ± 3% ^Cbc^ | 99.8 ± 0.9% ^ABa^ | 93 ± 2% ^Bc^ | 46.0 ± 0.7% ^Cd^ | 95 ± 1% ^Bbc^ | 46.0 ± 0.1% ^Cd^ |
| 42 | - | - | - | - | 91.2 ± 0.9% ^Ba^ | 43.40 ± 0.9% ^Db^ | 89 ± 3% ^Ca^ | 44.2 ± 0.1% ^Db^ |
| 63 | 100.5 ± 0.6% ^ABa^ | 103.0 ± 0.7% ^Aa^ | 100. ± 3% ^ABCa^ | 104 ± 2% ^Aa^ | 85 ± 3% ^CDb^ | 41.42 ± 0.6% ^Ec^ | 86 ± 2% ^Cb^ | 42.2 ± 0.2% ^Ec^ |
| 91 | 101 ± 1% ^ABab^ | 99.6 ± 0.4% ^ABb^ | 104 ± 3% ^Aa^ | 103 ± 1% ^Aab^ | 89 ± 3% ^BCc^ | 38.7 ± 0.5% ^Fd^ | 85.2 ± 0.5% ^CDc^ | 39.2 ± 0.2% ^Fd^ |
| 119 | 101 ± 2% ^Aa^ | 101 ± 3% ^ABa^ | 100. ± 1% ^ABCa^ | 101 ± 2% ^ABa^ | 82.4 ± 0.3% ^DEb^ | 37.7 ± 0.2% ^FGc^ | 81.5 ± 0.2% ^DEb^ | 38.4 ± 0.3% ^FGc^ |
| 140 | 100. ± 2% ^ABa^ | 99 ± 3% ^ABa^ | 100. ± 1% ^ABCa^ | 101 ± 3% ^ABa^ | 79.0 ± 0.3% ^Eb^ | 36.7 ± 0.1% ^Gc^ | 78.8 ± 0.3% ^Eb^ | 37.5 ± 0.3% ^GHc^ |
| 161 | 101 ± 2% ^ABa^ | 99 ± 2% ^ABa^ | 101 ± 1% ^ABa^ | 101 ± 2% ^ABa^ | 78.6 ± 0.2% ^Eb^ | 36.6 ± 0.2% ^Gc^ | 78.5 ± 0.1% ^Eb^ | 37.4 ± 0.4% ^Hc^ |
| 392 | 96 ± 3% ^Ba^ | 99.8 ± 0.4% ^ABa^ | 98 ± 2% ^BCa^ | 101.3 ± 0.2% ^ABa^ | 57 ± 3% ^Fb^ | 32.1 ± 0.1% ^Hc^ | 59 ± 3% ^Fb^ | 33.1 ± 0.4% ^Ic^ |

**C)**

| **TClHCl 60ºC**  **Percent Vitamin Remaining** | | | | | | | | |
| --- | --- | --- | --- | --- | --- | --- | --- | --- |
| **Days** | **pH 3** | | | | **pH 6** | | | |
|  | **TClHCl with HCl** | | **TClHCl with HNO_3_** | | **TClHCl with HCl** | | **TClHCl with HNO_3_** | |
|  | **1 mg/mL** | **20 mg/mL** | **1 mg/mL** | **20 mg/mL** | **1 mg/mL** | **20 mg/mL** | **1 mg/mL** | **20 mg/mL** |
| 0 | 100.0 ± 0.4% ^Aa^ | 100.1 ± 0.6% ^Aa^ | 100.1 ± 0.3% ^Aa^ | 100.0 ± 0.2% ^Aa^ | 100.1 ± 0.4% ^Aa^ | 99.9 ± 0.4% ^Aa^ | 100. ± 1% ^Aa^ | 100.1 ± 0.3% ^Aa^ |
| 1 | - | - | - | - | 99.3 ± 0.3% ^Aa^ | 96.1 ± 0.2% ^Bb^ | 99.6 ± 0.2% ^Aa^ | 96.3 ± 0.4% ^Bb^ |
| 2 | - | - | - | - | - | 79.0 ± 0.6% ^Ca^ | - | 79.3 ± 0.4% ^Ca^ |
| 3 | - | - | - | - | 85.1 ± 0.4% ^Bb^ | 45.6 ± 0.4% ^Dc^ | 86.5 ± 0.6% ^Ba^ | 45.1 ± 0.3% ^Dc^ |
| 7 | 99.0 ± 0.2% ^ABa^ | 100.0 ± 0.9% ^Aa^ | 99.3 ± 0.5% ^Aa^ | 99.1 ± 0.4% ^Aa^ | 83.4 ± 0.2% ^Cb^ | 42.1 ± 0.3% ^Ec^ | 84.5 ± 0.3% ^Cb^ | 42.0 ± 0.2% ^Ec^ |
| 12 | - | - | - | - | 75.67 ± 0.09% ^Db^ | 39.4 ± 0.2% ^Fc^ | 76.6 ± 0.1% ^Da^ | 39.0 ± 0.3% ^Fc^ |
| 16 | - | - | - | - | 69.1 ± 0.1% ^Ea^ | 37.80 ± 0.06% ^Gb^ | 69.6 ± 0.3% ^Ea^ | 38.5 ± 0.2% ^Gb^ |
| 21 | - | - | - | - | 66.7 ± 0.4% ^Fa^ | 36.6 ± 0.3% ^Hb^ | 67.6 ± 0.1% ^Fa^ | 36.0 ± 0.5% ^Hb^ |
| 28 | 95.0 ± 0.6% ^Ba^ | 97 ± 1% ^Aa^ | 98 ± 3% ^Aa^ | 95 ± 4% ^Aa^ | - | - | - | - |
| 63 | 88 ± 2% ^Cb^ | 97 ± 3% ^Aa^ | 92 ± 2% ^Bab^ | 97 ± 1% ^Aa^ | - | - | - | - |
| 91 | 78 ± 2% ^Da^ | 84 ± 4% ^Aa^ | 83 ± 1% ^Ca^ | 82 ± 1% ^Ba^ | - | - | - | - |
| 119 | 71 ± 2% ^Ea^ | 75 ± 5% ^Ca^ | 74.2 ± 0.4% ^Da^ | 74 ± 2% ^Ca^ | - | - | - | - |
| 140 | 67 ± 2% ^EFa^ | 72 ± 4% ^CDa^ | 71.0 ± 0.6% ^Da^ | 70. ± 1% ^CDa^ | - | - | - | - |
| 161 | 63 ± 2% ^Fa^ | 66 ± 4% ^Da^ | 67.1 ± 0.6% ^Ea^ | 65.4 ± 0.3% ^Da^ | - | - | - | - |
| 392 | 35 ± 2% ^Ga^ | 38 ± 3% ^Ea^ | 39 ± 1% ^Fa^ | 36 ± 2% ^Ea^ | - | - | - | - |

**D)**

| **TClHCl 70ºC**  **Percent Vitamin Remaining** | | | | | | | | |
| --- | --- | --- | --- | --- | --- | --- | --- | --- |
| **Days** | **pH 3** | | | | **pH 6** | | | |
|  | **TClHCl with HCl** | | **TClHCl with HNO_3_** | | **TClHCl with HCl** | | **TClHCl with HNO_3_** | |
|  | **1 mg/mL** | **20 mg/mL** | **1 mg/mL** | **20 mg/mL** | **1 mg/mL** | **20 mg/mL** | **1 mg/mL** | **20 mg/mL** |
| 0 | 100.1 ± 0.4% ^Aa^ | 100. ± 1% ^Aa^ | 100.0 ± 0.2% ^Aa^ | 99.9 ± 0.8% ^Aa^ | 100.1 ± 0.4% ^Aa^ | 99.9 ± 0.4% ^Aa^ | 100. ± 1% ^Aa^ | 100.1 ± 0.3% ^Aa^ |
| 1 | 99.9 ± 0.7% ^Aa^ | 100.1 ± 0.2% ^Aa^ | 100. ± 3% ^Aa^ | 101.1 ± 0.5% ^Aa^ | 91 ± 1% ^Bb^ | 55 ± 1% ^Bc^ | 92 ± 1% ^Bb^ | 53.6 ± 0.7% ^Bc^ |
| 2 | 99.9 ± 0.2% ^Aa^ | 100.3 ± 0.5% ^Aa^ | 100. ± 1% ^Aa^ | 101.2 ± 0.2% ^Aa^ | 87.2 ± 0.8% ^Cb^ | 46.4 ± 0.3% ^Cc^ | 87.8 ± 0.7% ^Cb^ | 46.1 ± 0.1% ^Cc^ |
| 4 | 99 ± 2% ^Aa^ | 99 ± 1% ^Aa^ | 100.3 ± 0.4% ^Aa^ | 100.2 ± 0.4% ^Aa^ | 78.5 ± 0.7% ^Db^ | 41.6 ± 0.1% ^Dc^ | 79.0 ± 0.7% ^Db^ | 41.5 ± 0.1% ^Dc^ |
| 7 | 98 ± 2% ^ABa^ | 98.4 ± 0.6% ^Aa^ | 99.4 ± 0.7% ^Aa^ | 100. ± 1% ^Aa^ | 68.7 ± 0.8% ^Eb^ | 38.1 ± 0.2% ^Ec^ | 69.1 ± 0.1% ^Eb^ | 37.88 ± 0.07% ^Ec^ |
| 13 | 94 ± 2% ^Bb^ | 95.3 ± 0.8% ^Bab^ | 98 ± 2% ^Aa^ | 97 ± 1% ^Bab^ | 53.4 ± 0.7% ^Fc^ | 32.6 ± 0.3% ^Fd^ | 53.8 ± 0.2% ^Fc^ | 32.5 ± 0.3% ^Fd^ |
| 29 | 81 ± 4% ^Ca^ | 86 ± 1% ^Ca^ | 85 ± 3% ^Ba^ | 85 ± 1% ^Ca^ | - | - | - | - |
| 42 | 69.0 ± 0.8% ^Db^ | 75 ± 1% ^Da^ | 75.4 ± 0.3% ^Ca^ | 76 ± 1% ^Da^ | - | - | - | - |

**E)**

| **TClHCl 80ºC**  **Percent Vitamin Remaining** | | | | | | | | |
| --- | --- | --- | --- | --- | --- | --- | --- | --- |
| **Days** | **pH 3** | | | | **pH 6** | | | |
|  | **TClHCl with HCl** | | **TClHCl with HNO_3_** | | **TClHCl with HCl** | | **TClHCl with HNO_3_** | |
|  | **1 mg/mL** | **20 mg/mL** | **1 mg/mL** | **20 mg/mL** | **1 mg/mL** | **20 mg/mL** | **1 mg/mL** | **20 mg/mL** |
| 0 | 98.8 ± 0.5% ^Abc^ | 96.84 ± 0.05% ^Ad^ | 99.7 ± 0.6% ^Aab^ | 99.7 ± 0.8% ^Aab^ | 100.04 ± 0.08% ^Aab^ | 97.8 ± 0.4% ^Acd^ | 100.6 ± 0.5% ^Aa^ | 99.2 ± 0.7% ^Aabc^ |
| 1 | 98.0 ± 0.8% ^Aa^ | 98.8 ± 0.2% ^Aa^ | 96.9 ± 0.8% ^ABa^ | 97.5 ± 0.1% ^Aa^ | 86.5 ± 0.3% ^Bb^ | 49 ± 1% ^Bc^ | 85 ± 2% ^Bb^ | 46.1 ± 0.8% ^Bc^ |
| 2 | - | - | - | - | 74.4 ± 0.6% ^Cb^ | 42 ± 3% ^Bc^ | 81 ± 2% ^Ba^ | 42 ± 2% ^Cc^ |
| 4 | 89.7 ± 0.7% ^Bb^ | 89 ± 2% ^Bb^ | 93 ± 2% ^BCab^ | 94.3 ± 0.1% ^Ba^ | 57.7 ± 0.7% ^Dc^ | 35 ± 3% ^Cd^ | 58.1 ± 0.6% ^Cc^ | 35 ± 2% ^Dd^ |
| 7 | 86 ± 4% ^Ba^ | 89 ± 1% ^Ba^ | 89 ± 4% ^Ca^ | 91.8 ± 0.8% ^Ba^ | 42.8 ± 0.9% ^Ec^ | 30. ± 3% ^CDd^ | 51 ± 4% ^Db^ | 29 ± 2% ^Ed^ |
| 13 | 73 ± 2% ^Cc^ | 75 ± 1% ^Cbc^ | 79.3 ± 0.4% ^Dab^ | 81 ± 1% ^Ca^ | 24.6 ± 0.6% ^Fe^ | 25 ± 4% ^De^ | 41 ± 2% ^Ed^ | 23 ± 1% ^Fe^ |
| 29 | 48.9 ± 0.4% ^Dc^ | 56 ± 2% ^Db^ | 60. ± 3% ^Eab^ | 63 ± 2% ^Da^ | - | - | - | - |

**Additional Table S3.** pH of TMN solutions after storage at the specified conditions over time: **A)** 25ºC, **B)** 40ºC, **C)** 60ºC, **D)** 70ºC, and **E)** 80ºC. Uppercase superscript letters on values for each sample type denote statistical significance within that sample type (down columns). Lowercase superscript letters denote statistical significance between sample types for each day (across rows).

**A)**

| **TMN 25ºC**  **pH Over Time** | | | | | | | | |
| --- | --- | --- | --- | --- | --- | --- | --- | --- |
| **Days** | **pH 3** | | | | **pH 6** | | | |
|  | **TMN with HNO_3_** | | **TMN with HCl** | | **TMN with HNO_3_** | | **TMN with HCl** | |
|  | **1 mg/mL** | **20 mg/mL** | **1 mg/mL** | **20 mg/mL** | **1 mg/mL** | **20 mg/mL** | **1 mg/mL** | **20 mg/mL** |
| 0 | 3 ^BCb^ | 3 ^Bb^ | 3 ^Db^ | 3 ^Ab^ | 6 ^Aa^ | 6 ^Ba^ | 6 ^Aa^ | 6 ^Ba^ |
| 7 | 3.042 ± 0.003 ^Ad^ | 3.008 ± 0.003 ^Be^ | 3.036 ± 0.004 ^Cd^ | 2.999 ± 0.003 ^Ae^ | 5.913 ± 0.004 ^Ac^ | 6.033 ± 0.004 ^Aa^ | 5.965 ± 0.004 ^Bb^ | 6.020 ± 0.003 ^Aa^ |
| 63 | 2.98 ± 0.02 ^Ce^ | 2.966 ± 0.004 ^Ce^ | 3.024 ± 0.005 ^Cd^ | 2.992 ± 0.004 ^ABde^ | 5.7105 ± 0.0007 ^Bb^ | 5.444 ± 0.006 ^Cc^ | 5.753 ± 0.004 ^Ca^ | 5.468 ± 0.004 ^Cc^ |
| 161 | 3.033 ± 0.003 ^ABf^ | 3.033 ± 0.002 ^Af^ | 3.096 ± 0.004 ^Be^ | 2.997 ± 0.008 ^Ag^ | 5.315 ± 0.004 ^Ca^ | 4.963 ± 0.004 ^Dc^ | 5.285 ± 0.004 ^Db^ | 4.905 ± 0.003 ^Dd^ |
| 392 | 3.035 ± 0.004 ^ABd^ | 2.998 ± 0.003 ^Bd^ | 3.144 ± 0.004 ^Ac^ | 2.975 ± 0.005 ^Bd^ | 4.93 ± 0.05 ^Db^ | 4.914 ± 0.002 ^Eb^ | 5.078 ± 0.006 ^Ea^ | 4.911 ± 0.004 ^Db^ |

**B)**

| **TMN 40ºC**  **pH Over Time** | | | | | | | | |
| --- | --- | --- | --- | --- | --- | --- | --- | --- |
| **Days** | **pH 3** | | | | **pH 6** | | | |
|  | **TMN with HNO_3_** | | **TMN with HCl** | | **TMN with HNO_3_** | | **TMN with HCl** | |
|  | **1 mg/mL** | **20 mg/mL** | **1 mg/mL** | **20 mg/mL** | **1 mg/mL** | **20 mg/mL** | **1 mg/mL** | **20 mg/mL** |
| 0 | 3 ^CBb^ | 3 ^Ab^ | 3 ^Cb^ | 3 ^Ab^ | 6 ^Aa^ | 6 ^Aa^ | 6 ^Aa^ | 6 ^Aa^ |
| 7 | 3.055 ± 0.005 ^Ac^ | 3.00 ± 0.01 ^Ac^ | 2.99 ± 0.01 ^Cc^ | 2.98 ± 0.01 ^ABc^ | 5.740 ± 0.002 ^Bb^ | 5.891 ± 0.003 ^Ba^ | 5.76 ± 0.03 ^Bb^ | 5.95 ± 0.05 ^Aa^ |
| 63 | 3.02 ± 0.02 ^ABc^ | 2.942 ± 0.004 ^Bd^ | 3.026 ± 0.005 ^Bc^ | 2.94 ± 0.01 ^Cd^ | 5.220 ± 0.003 ^Ca^ | 4.920 ± 0.004 ^Cb^ | 5.248 ± 0.006 ^Ca^ | 4.913 ± 0.008 ^Bb^ |
| 161 | 3.027 ± 0.005 ^ABd^ | 2.945 ± 0.002 ^Be^ | 3.078 ± 0.004 ^Ac^ | 2.959 ± 0.004 ^BCe^ | 5.110 ± 0.004 ^Da^ | 4.90 ± 0.01 ^Cb^ | 5.120 ± 0.002 ^Da^ | 4.901 ± 0.004 ^Bb^ |
| 392 | 2.970 ± 0.005 ^Cf^ | 2.780 ± 0.002 ^Cg^ | 3.009 ± 0.003 ^BCe^ | 2.749 ± 0.004 ^Dh^ | 4.965 ± 0.003 ^Eb^ | 4.824 ± 0.003 ^Dd^ | 4.999 ± 0.003 ^Ea^ | 4.88 ± 0.02 ^Bc^ |

**C)**

| **TMN 60ºC**  **pH Over Time** | | | | | | | | |
| --- | --- | --- | --- | --- | --- | --- | --- | --- |
| **Days** | **pH 3** | | | | **pH 6** | | | |
|  | **TMN with HNO_3_** | | **TMN with HCl** | | **TMN with HNO_3_** | | **TMN with HCl** | |
|  | **1 mg/mL** | **20 mg/mL** | **1 mg/mL** | **20 mg/mL** | **1 mg/mL** | **20 mg/mL** | **1 mg/mL** | **20 mg/mL** |
| 0 | 3 ^Ab^ | 3 ^Ab^ | 3 ^Ab^ | 3 ^Ab^ | 6 ^Ba^ | 6 ^Aa^ | 6 ^Ba^ | 6 ^Aa^ |
| 1 | - | - | - | - | 6.23 ± 0.09 ^Aa^ | 5.8445 ± 0.0007 ^Bb^ | 6.106 ± 0.003 ^Aa^ | 5.823 ± 0.005 ^Bb^ |
| 3 | - | - | - | - | 5.676 ± 0.003 ^Ca^ | 5.039 ± 0.005 ^Cc^ | 5.654 ± 0.006 ^Cb^ | 5.041 ± 0.004 ^Cc^ |
| 7 | 3.02 ± 0.01 ^Aa^ | 2.939 ± 0.008 ^Ab^ | 3.01 ± 0.01 ^Aa^ | 2.921 ± 0.002 ^Bb^ | - | - | - | - |
| 12 | - | - | - | - | 5.356 ± 0.003 ^Da^ | 4.931 ± 0.004 ^Db^ | 5.376 ± 0.004 ^Da^ | 4.86 ± 0.05 ^Db^ |
| 21 | - | - | - | - | 5.270 ± 0.003 ^Da^ | 4.920 ± 0.003 ^Dc^ | 5.241 ± 0.004 ^Eb^ | 4.903 ± 0.002 ^Dd^ |
| 63 | 2.956 ± 0.004 ^Aa^ | 2.587 ± 0.003 ^Bc^ | 2.941 ± 0.002 ^Ab^ | 2.573 ± 0.002 ^Cd^ | - | - | - | - |
| 161 | 2.49 ± 0.06 ^Bb^ | 2.19 ± 0.09 ^Cc^ | 2.84 ± 0.04 ^Ba^ | 2.310 ± 0.009 ^Dbc^ | - | - | - | - |
| 392 | 2.9 ± 0.1 ^Aa^ | 2.0 ± 0.1 ^Cb^ | - | 2.210 ± 0.003 ^Eb^ | - | - | - | - |

| **TMN 70ºC**  **pH Over Time** | | | | | | | | |
| --- | --- | --- | --- | --- | --- | --- | --- | --- |
| **Days** | **pH 3** | | | | **pH 6** | | | |
|  | **TMN with HNO_3_** | | **TMN with HCl** | | **TMN with HNO_3_** | | **TMN with HCl** | |
|  | **1 mg/mL** | **20 mg/mL** | **1 mg/mL** | **20 mg/mL** | **1 mg/mL** | **20 mg/mL** | **1 mg/mL** | **20 mg/mL** |
| 0 | 3 ^Bb^ | 3 ^Ab^ | 3 ^ABb^ | 3 ^Ab^ | 6 ^Aa^ | 6 ^Aa^ | 6 ^Aa^ | 6 ^Aa^ |
| 1 | 3.00 ± 0.01 ^Bcd^ | 2.99 ± 0.01 ^Acd^ | 3.034 ± 0.009 ^Ac^ | 2.8 ± 0.1 ^ABd^ | 5.69 ± 0.02 ^Ba^ | 5.173 ± 0.007 ^Bb^ | 5.7195 ± 0.0007 ^Ba^ | 5.140 ± 0.003 ^Bb^ |
| 4 | 3.075 ± 0.006 ^Ac^ | 2.977 ± 0.006 ^Ad^ | 2.997 ± 0.006 ^ABd^ | 2.893 ± 0.005 ^ABe^ | 5.47 ± 0.01 ^Ca^ | 5.063 ± 0.006 ^Cb^ | 5.460 ± 0.004 ^Ca^ | 5.06 ± 0.02 ^Cb^ |
| 13 | 2.930 ± 0.003 ^Ce^ | 2.828 ± 0.003 ^Bf^ | 2.99 ± 0.02 ^Bd^ | 2.804 ± 0.002 ^Bf^ | 5.066 ± 0.002 ^Db^ | 4.816 ± 0.003 ^Dc^ | 5.120 ± 0.004 ^Da^ | 4.831 ± 0.004 ^Dc^ |
| 29 | 2.895 ± 0.004 ^Db^ | 2.562 ± 0.003 ^Cc^ | 2.922 ± 0.003 ^Ca^ | 2.565 ± 0.006 ^Cc^ | - | - | - | - |
| 42 | 2.897 ± 0.004 ^Db^ | 2.5745 ± 0.0007 ^Cc^ | 2.9275 ± 0.0007 ^Ca^ | 2.517 ± 0.004 ^Cd^ | - | - | - | - |

**D)**

**E)**

| **TMN 80ºC**  **pH Over Time** | | | | | | | | |
| --- | --- | --- | --- | --- | --- | --- | --- | --- |
| **Days** | **pH 3** | | | | **pH 6** | | | |
|  | **TMN with HNO_3_** | | **TMN with HCl** | | **TMN with HNO_3_** | | **TMN with HCl** | |
|  | **1 mg/mL** | **20 mg/mL** | **1 mg/mL** | **20 mg/mL** | **1 mg/mL** | **20 mg/mL** | **1 mg/mL** | **20 mg/mL** |
| 0 | 3 ^BCb^ | 3 ^Ab^ | 3 ^Bb^ | 3 ^Ab^ | 6 ^Aa^ | 6 ^Aa^ | 6 ^Aa^ | 6 ^Aa^ |
| 1 | 3.20 ± 0.02 ^Ae^ | 3.03 ± 0.01 ^Af^ | 2.98 ± 0.04 ^BCf^ | 2.77 ± 0.02 ^Bg^ | 5.18 ± 0.06 ^Cb^ | 4.63 ± 0.02 ^Dd^ | 5.56 ± 0.04 ^Ba^ | 5.00 ± 0.04 ^Bc^ |
| 2 | 3.092 ± 0.001 ^ABe^ | 2.965 ± 0.004 ^Af^ | 3.158 ± 0.004 ^Ad^ | 2.960 ± 0.004 ^Af^ | 5.699 ± 0.005 ^Ba^ | 5.080 ± 0.004 ^Bc^ | 5.624 ± 0.004 ^Bb^ | 5.092 ± 0.004 ^Bc^ |
| 4 | 3.02 ± 0.03 ^ABCc^ | 2.70 ± 0.03 ^Be^ | 2.92 ± 0.03 ^BCd^ | 2.64 ± 0.01 ^Ce^ | 5.18 ± 0.03 ^Ca^ | 4.83 ± 0.02 ^Cb^ | 5.26 ± 0.01 ^Ca^ | 4.845 ± 0.007 ^Cb^ |
| 7 | 3.09 ± 0.05 ^ABc^ | 2.62 ± 0.03 ^Bd^ | 2.96 ± 0.03 ^BCc^ | 2.61 ± 0.03 ^Cd^ | 5.11 ± 0.05 ^Ca^ | 4.82 ± 0.03 ^Cb^ | 5.22 ± 0.02 ^Ca^ | 4.78 ± 0.06 ^Cb^ |
| 13 | 2.99 ± 0.08 ^BCc^ | 2.41 ± 0.02 ^Cd^ | 2.94 ± 0.02 ^BCc^ | 2.42 ± 0.03 ^Dd^ | 4.92 ± 0.02 ^Da^ | 4.63 ± 0.04 ^Db^ | 4.97 ± 0.04 ^Da^ | 4.62 ± 0.02 ^Db^ |
| 29 | 2.89 ± 0.06 ^Ca^ | 2.30 ± 0.03 ^Db^ | 2.87 ± 0.04 ^Ca^ | 2.30 ± 0.01 ^Eb^ | - | - | - | - |

**Additional Table S4.** pH of TClHCl solutions after storage at the specified conditions over time: **A)** 25ºC, **B)** 40ºC, **C)** 60ºC, **D)** 70ºC, and **E)** 80ºC. Uppercase superscript letters on values for each sample type denote statistical significance within that sample type (down columns). Lowercase superscript letters denote statistical significance between sample types for each day (across rows).

**A)**

| **TClHCl 25ºC**  **pH Over Time** | | | | | | | | |
| --- | --- | --- | --- | --- | --- | --- | --- | --- |
| **Days** | **pH 3** | | | | **pH 6** | | | |
|  | **TClHCl with HCl** | | **TClHCl with HNO_3_** | | **TClHCl with HCl** | | **TClHCl with HNO_3_** | |
|  | **1 mg/mL** | **20 mg/mL** | **1 mg/mL** | **20 mg/mL** | **1 mg/mL** | **20 mg/mL** | **1 mg/mL** | **20 mg/mL** |
| 0 | 3 ^Cb^ | 3 ^Cb^ | 3 ^Bb^ | 3 ^Db^ | 6 ^Ba^ | 6 ^Ba^ | 6 ^Ca^ | 6 ^Ba^ |
| 7 | 3.067 ± 0.004 ^Ad^ | 3.036 ± 0.005 ^Be^ | 3.003 ± 0.004 ^Bf^ | 3.076 ± 0.005 ^Ad^ | 6.169 ± 0.003 ^Ab^ | 6.073 ± 0.004 ^Ac^ | 6.245 ± 0.003 ^Aa^ | 6.081 ± 0.004 ^Ac^ |
| 63 | 3.050 ± 0.004 ^ABe^ | 3.035 ± 0.005 ^Bef^ | 3.037 ± 0.006 ^Aef^ | 3.025 ± 0.005 ^Cf^ | 5.965 ± 0.004 ^Cb^ | 5.905 ± 0.005 ^Cc^ | 6.038 ± 0.004 ^Ba^ | 5.862 ± 0.002 ^Cd^ |
| 161 | 3.042 ± 0.005 ^Bd^ | 3.007 ± 0.003 ^Cf^ | 3.038 ± 0.004 ^Ad^ | 3.024 ± 0.004 ^Ce^ | 5.240 ± 0.004 ^Db^ | 5.045 ± 0.003 ^Dc^ | 5.396 ± 0.002 ^Da^ | 5.042 ± 0.003 ^Dc^ |
| 392 | 3.058 ± 0.006 ^ABd^ | 3.054 ± 0.004 ^Ad^ | 2.998 ± 0.003 ^Be^ | 3.049 ± 0.004 ^Bd^ | 4.980 ± 0.004 ^Ec^ | 5.03 ± 0.01 ^Db^ | 5.066 ± 0.004 ^Ea^ | 4.9875 ± 0.0007 ^Ec^ |

**B)**

| **TClHCl 40ºC**  **pH Over Time** | | | | | | | | |
| --- | --- | --- | --- | --- | --- | --- | --- | --- |
| **Days** | **pH 3** | | | | **pH 6** | | | |
|  | **TClHCl with HCl** | | **TClHCl with HNO_3_** | | **TClHCl with HCl** | | **TClHCl with HNO_3_** | |
|  | **1 mg/mL** | **20 mg/mL** | **1 mg/mL** | **20 mg/mL** | **1 mg/mL** | **20 mg/mL** | **1 mg/mL** | **20 mg/mL** |
| 0 | 3 ^Ab^ | 3 ^Ab^ | 3 ^Cb^ | 3 ^Bb^ | 6 ^Aa^ | 6 ^Aa^ | 6 ^Ba^ | 6 ^Aa^ |
| 7 | 2.96 ± 0.07 ^Ac^ | 3.02 ± 0.01 ^Ac^ | 3.007 ± 0.004 ^BCc^ | 3.035 ± 0.002 ^Ac^ | 6.009 ± 0.003 ^Aa^ | 5.926 ± 0.005 ^Bab^ | 6.019 ± 0.004 ^Aa^ | 5.910 ± 0.008 ^Bb^ |
| 63 | 2.993 ± 0.004 ^Ae^ | 2.976 ± 0.004 ^Bf^ | 3.0115 ± 0.0007 ^BCd^ | 2.984 ± 0.004 ^Cef^ | 5.305 ± 0.003 ^Bb^ | 4.987 ± 0.004 ^Cc^ | 5.345 ± 0.003 ^Ca^ | 4.994 ± 0.004 ^Cc^ |
| 161 | 3.048 ± 0.004 ^Ae^ | 2.946 ± 0.004 ^Cg^ | 3.034 ± 0.004 ^Ae^ | 2.983 ± 0.004 ^Cf^ | 5.125 ± 0.004 ^Cb^ | 4.940 ± 0.003 ^Dc^ | 5.147 ± 0.009 ^Da^ | 4.896 ± 0.004 ^Dd^ |
| 392 | 3.046 ± 0.004 ^Ae^ | 2.769 ± 0.001 ^Dh^ | 3.019 ± 0.004 ^Bf^ | 2.825 ± 0.002 ^Dg^ | 4.942 ± 0.004 ^Db^ | 4.837 ± 0.004 ^Ed^ | 4.9805 ± 0.0007 ^Ea^ | 4.882 ± 0.006 ^Dc^ |

**C)**

| **TClHCl 60ºC**  **pH Over Time** | | | | | | | | |
| --- | --- | --- | --- | --- | --- | --- | --- | --- |
| **Days** | **pH 3** | | | | **pH 6** | | | |
|  | **TClHCl with HCl** | | **TClHCl with HNO_3_** | | **TClHCl with HCl** | | **TClHCl with HNO_3_** | |
|  | **1 mg/mL** | **20 mg/mL** | **1 mg/mL** | **20 mg/mL** | **1 mg/mL** | **20 mg/mL** | **1 mg/mL** | **20 mg/mL** |
| 0 | 3 ^Bb^ | 3 ^Ab^ | 3 ^ABb^ | 3 ^Ab^ | 6 ^Ba^ | 6 ^Aa^ | 6 ^Ba^ | 6 ^Aa^ |
| 1 | - | - | - | - | 6.130 ± 0.003 ^Ab^ | 5.904 ± 0.004 ^Bd^ | 6.178 ± 0.005 ^Aa^ | 5.98 ± 0.01 ^Ac^ |
| 3 | - | - | - | - | 5.745 ± 0.009 ^Ca^ | 5.12 ± 0.03 ^Cb^ | 5.772 ± 0.004 ^Ca^ | 5.104 ± 0.004 ^Bb^ |
| 7 | 3.041 ± 0.004 ^Aa^ | 2.985 ± 0.004 ^Ab^ | 3.033 ± 0.003 ^Aa^ | 2.957 ± 0.002 ^Ac^ | - | - | - | - |
| 12 | - | - | - | - | 5.419 ± 0.006 ^Db^ | 4.981 ± 0.005 ^Dc^ | 5.458 ± 0.006 ^Da^ | 4.983 ± 0.006 ^Cc^ |
| 21 | - | - | - | - | 5.292 ± 0.003 ^Eb^ | 4.963 ± 0.003 ^Dd^ | 5.317 ± 0.001 ^Ea^ | 4.980 ± 0.004 ^Cc^ |
| 63 | 2.982 ± 0.004 ^Ba^ | 2.782 ± 0.005 ^Bc^ | 2.962 ± 0.004 ^BCb^ | 2.770 ± 0.004 ^Bd^ | - | - | - | - |
| 161 | 2.86 ± 0.01 ^Ca^ | 2.29 ± 0.02 ^Cb^ | 2.91 ± 0.04 ^CDa^ | 2.29 ± 0.02 ^Cb^ | - | - | - | - |
| 392 | 2.850 ± 0.004 ^Cb^ | 2.258 ± 0.004 ^Cc^ | 2.870 ± 0.004 ^Da^ | 2.240 ± 0.004 ^Dd^ | - | - | - | - |

**D)**

| **TClHCl 70ºC**  **pH Over Time** | | | | | | | | |
| --- | --- | --- | --- | --- | --- | --- | --- | --- |
| **Days** | **pH 3** | | | | **pH 6** | | | |
|  | **TClHCl with HCl** | | **TClHCl with HNO_3_** | | **TClHCl with HCl** | | **TClHCl with HNO_3_** | |
|  | **1 mg/mL** | **20 mg/mL** | **1 mg/mL** | **20 mg/mL** | **1 mg/mL** | **20 mg/mL** | **1 mg/mL** | **20 mg/mL** |
| 0 | 3 ^Bb^ | 3 ^Ab^ | 3 ^Bb^ | 3 ^Ab^ | 6 ^Aa^ | 6 ^Aa^ | 6 ^Aa^ | 6 ^Aa^ |
| 1 | 2.99 ± 0.02 ^Bc^ | 2.99 ± 0.04 ^Ac^ | 2.974 ± 0.006 ^Cc^ | 3.004 ± 0.003 ^Ac^ | 5.78 ± 0.04 ^Ba^ | 5.27 ± 0.01 ^Bb^ | 5.80 ± 0.02 ^Ba^ | 5.21 ± 0.01 ^Bb^ |
| 4 | 3.039 ± 0.004 ^Ae^ | 2.990 ± 0.004 ^Af^ | 3.055 ± 0.005 ^Ae^ | 3.008 ± 0.005 ^Af^ | 5.556 ± 0.009 ^Ca^ | 5.052 ± 0.004 ^Cd^ | 5.533 ± 0.001 ^Cb^ | 5.075 ± 0.004 ^Cc^ |
| 13 | 3.032 ± 0.004 ^Ac^ | 2.869 ± 0.002 ^Be^ | 2.96 ± 0.01 ^Cd^ | 2.84 ± 0.02 ^Be^ | 5.155 ± 0.003 ^Da^ | 4.899 ± 0.005 ^Db^ | 5.159 ± 0.003 ^Da^ | 4.9075 ± 0.004 ^Db^ |
| 29 | 2.923 ± 0.003 ^Ca^ | 2.712 ± 0.003 ^Cd^ | 2.887 ± 0.002 ^Db^ | 2.775 ± 0.004 ^Cc^ | - | - | - | - |
| 42 | 2.975 ± 0.001 ^Ba^ | 2.70 ± 0.01 ^Cd^ | 2.885 ± 0.004 ^Db^ | 2.752 ± 0.004 ^Cc^ | - | - | - | - |

**E)**

| **TClHCl 80ºC**  **pH Over Time** | | | | | | | | |
| --- | --- | --- | --- | --- | --- | --- | --- | --- |
| **Days** | **pH 3** | | | | **pH 6** | | | |
|  | **TClHCl with HCl** | | **TClHCl with HNO_3_** | | **TClHCl with HCl** | | **TClHCl with HNO_3_** | |
|  | **1 mg/mL** | **20 mg/mL** | **1 mg/mL** | **20 mg/mL** | **1 mg/mL** | **20 mg/mL** | **1 mg/mL** | **20 mg/mL** |
| 0 | 3 ^Ab^ | 3 ^Ab^ | 3 ^ABb^ | 3 ^Ab^ | 6 ^Aa^ | 6 ^Aa^ | 6 ^Aa^ | 6 ^Aa^ |
| 1 | 3.12 ± 0.03 ^Ae^ | 2.93 ± 0.01 ^ABf^ | 3.02 ± 0.02 ^ABf^ | 2.82 ± 0.01 ^Cg^ | 5.63 ± 0.04 ^Ba^ | 5.01 ± 0.03 ^Bc^ | 5.52 ± 0.03 ^Bb^ | 4.81 ± 0.02 ^Cd^ |
| 2 | 3.108 ± 0.008 ^Ac^ | 2.919 ± 0.003 ^ABd^ | 3.11 ± 0.07 ^Ac^ | 2.892 ± 0.005 ^Bd^ | 5.65 ± 0.01 ^Ba^ | 5.061 ± 0.004 ^Bb^ | 5.65 ± 0.02 ^Ba^ | 5.022 ± 0.008 ^Bb^ |
| 4 | 3.11 ± 0.03 ^Ad^ | 2.82 ± 0.03 ^BCe^ | 3.02 ± 0.03 ^ABd^ | 2.73 ± 0.02 ^De^ | 5.32 ± 0.03 ^Ca^ | 4.92 ± 0.03 ^Cb^ | 5.23 ± 0.01 ^Ca^ | 4.82 ± 0.02 ^Cc^ |
| 7 | 3.1 ± 0.1 ^Ac^ | 2.75 ± 0.04 ^Cd^ | 3.00 ± 0.04 ^ABcd^ | 2.72 ± 0.02 ^Dd^ | 5.13 ± 0.04 ^Da^ | 4.80 ± 0.02 ^Db^ | 5.20 ± 0.08 ^Ca^ | 4.73 ± 0.02 ^Db^ |
| 13 | 3.1 ± 0.2 ^Ac^ | 2.52 ± 0.03 ^Dd^ | 2.84 ± 0.02 ^Ccd^ | 2.63 ± 0.02 ^Ed^ | 4.94 ± 0.01 ^Ea^ | 4.59 ± 0.01 ^Eb^ | 5.03 ± 0.02 ^Da^ | 4.53 ± 0.01 ^Eb^ |
| 29 | 3.0 ± 0.1 ^Aa^ | 2.38 ± 0.05 ^Eb^ | 2.92 ± 0.04 ^BCa^ | 2.51 ± 0.01 ^Fb^ | - | - | - | - |
